# Housekeeping gene validation for RT-qPCR studies on synovial fibroblasts derived from healthy and osteoarthritic patients with focus on mechanical loading

**DOI:** 10.1371/journal.pone.0225790

**Published:** 2019-12-06

**Authors:** Ute Nazet, Agnes Schröder, Susanne Grässel, Dominique Muschter, Peter Proff, Christian Kirschneck

**Affiliations:** 1 Department of Orthodontics, University Medical Centre of Regensburg, Regensburg, Germany; 2 Department of Orthopaedics, University of Regensburg and Centre for Biomedical Technology, BioPark, Regensburg, Germany; University of Helsinki, FINLAND

## Abstract

Selection of appropriate housekeeping genes is essential for the validity of data normalization in reverse transcription quantitative PCR (RT-qPCR). Synovial fibroblasts (SF) play a mediating role in the development and progression of osteoarthritis (OA) pathogenesis, but there is no information on reliable housekeeping genes available. Therefore the goal of this study was to identify a set of reliable housekeeping genes suitable for studies of mechanical loading on SF from healthy and OA patients. Nine genes were evaluated towards expression stability and ranked according their relative stability determined by four different mathematical procedures (geNorm, NormFinder, BestKeeper and comparative ΔC_q_). We observed that *RPLP0* (*ribosomal protein*, *large*, *P0*) and *EEF1A1* (*eukaryotic translation elongation factor 1 alpha 1*) turned out to be the genes with the most stable expression in SF from non-OA or OA patients treated with or without mechanical loading. According to geNorm two genes are sufficient for normalization throughout. Expression of one tested target gene varied considerably, if normalized to different candidate housekeeping genes. Our study provides a tool for accurate and valid housekeeping gene selection in gene expression experiments on SF from healthy and OA patients with and without mechanical loading in consistent with the MIQE (Minimum Information for Publication of Quantitative Real-Time PCR Experiments) guidelines and additionally demonstrates the impact of proper housekeeping gene selection on the expression of the gene of interest.

## Introduction

For gene expression analysis there are currently three methods available: RNA-Seq, microarray analysis and RT-qPCR. RNA-Seq and microarray analysis were used to analyse many different gene expression profiles [1–5]. In contrast to these both methods reverse transcriptase quantitative PCR (RT-qPCR) allows to analyse the impact of various experimental conditions on the expression of one single gene [6–9]. Therefore RT-qPCR is still the method of choice for gene expression evaluation in most areas of molecular biology. Carefully chosen housekeeping genes guarantee precise gene expression quantification by accurate and valid data normalization [6,8,10]. One major feature of an optimal housekeeping gene is a small variation in expression across various experimental setups and cell types [8,11]. There are many studies dealing with the variation of expression stability of housekeeping genes across different experimental conditions or cell types [11,12]. The usage of not validated housekeeping genes in RT-pPCR studies can lead to potential bias and misinterpretation of experimental outcomes. To achieve conformity in RT-pPCR data evaluation, MIQE guidelines (Minimum Information for Publication of Quantitative Real-Time PCR Experiments) were established several years ago [10]. Usage of these guidelines is proposed to enhance reproducibility of RT-qPCR results [10]. The choice of housekeeping genes has a direct impact on the results of target gene analysis by RT-qPCR. To investigate the stability of different housekeeping genes, some mathematical algorithms like geNorm [12], NormFinder [13], BestKeeper [14] and the comparative ΔC_q_ method [15,16] were developed.

The geNorm [12] algorithm calculates the mean pairwise variation of the C_q_ values of one tested housekeeping gene compared with all other tested genes and specifies that value as stability M. Housekeeping genes with elevated M values are suggested to have an higher pairwise variation. As enhanced pairwise variation correlates with instable expression ratios, housekeeping genes with higher M values are not ideal for normalization [12]. With geNorm it is also possible to define the minimum required amount of housekeeping genes for target gene normalization, as it computes the gene stability by average pairwise variation among internal control genes [12].

The NormFinder algorithm [13] calculates intra- and intergroup variation between the tested housekeeping genes. Furthermore it designates a conjoint stability value for each tested housekeeping gene applying a model-based approach [13]. In this mathematical algorithm increased gene expression stability is associated with decreasing stability values.

The comparative ΔC_q_ method evaluates housekeeping genes on the basis of the standard deviation of the average ΔC_q_ aberrations of each tested housekeeping gene to all other tested housekeeping genes. Therefore it collates relative housekeeping gene expression within groups of biological replicates and from all other tested housekeeping genes [15,16].

The BestKeeper [14] algorithm makes use of the standard deviation of mean C_q_ of each tested housekeeping gene and evaluates gene stability by pairwise bivariate correlations of C_q_ values of each gene using a “BestKeeper Index“. Stably expressed housekeeping genes are reported to have higher r values. Until now various studies were performed to assess appropriate housekeeping genes for different experimental setups and cell types, but there are still no reliable housekeeping genes for studies on human synovial fibroblasts published.

Synovial fibroblasts play a leading part in the maintenance of a healthy joint. The synovium encases articular joints throughout the human body and maintains the integrity of articular cartilage by regulating synovial fluid volume and composition producing lubricin and hyaluronic acid [17]. Among others, synovial fibroblasts make up the major cell population in the synovium of joints and play a critical mediating role in the development and progression of osteoarthritis (OA), since they are able to secrete proinflammatory cytokines [18] and express immune-receptors like toll-like receptors (TLR) [19,20]. The degenerative disease OA is associated with several afflictions such as chronic pain, articular cartilage degradation and subchondral bone remodelling as well as induced synovitis [21]. Aging, obesity, sport injuries, genetic predisposition [22] and mechanical overload [23,24] are reported to be risk factors for OA development. Also excessive mechanical loading on normal articular cartilage may lead to the development of OA by disruption of cartilage matrix homeostasis [24,25]. For several years, interest in the stromal-cell-like synovial fibroblasts has increased, as they were identified as key players in the innate immune-system-response, inflammation-related processes and intercellular actions and as principal performers involved in OA development and progression.

The study was designed to 1) identify a set of stably expressed housekeeping genes for human synovial fibroblasts derived from OA and non-OA patients, particularly in experiments with mechanical loading simulating OA pathogenesis and progression, 2) to assess the effects of different housekeeping genes used for normalization on target gene expression and 3) to compare various mathematical procedures used regarding their conformity. As mechanical stress loading is one major risk factor for the development of OA, we aimed to assess stable housekeeping genes for pressure application in both tested synovial fibroblast cell lines.

## Materials and methods

### In vitro cell culture experiment setup

Synovial fibroblasts from a healthy, non-OA patient were obtained directly from BioIVT (PCD-90-0645). Synovial fibroblasts from an OA patient were obtained and cultured from tissue to be discarded during knee surgery in the Department of Orthopedics at the University of Regensburg. The study was approved by the Ethics Committee of the Faculty of Medicine Regensburg (approval ID 12-170-0150) and written informed consent was obtained from the tissue donor.

**Fig 1:**
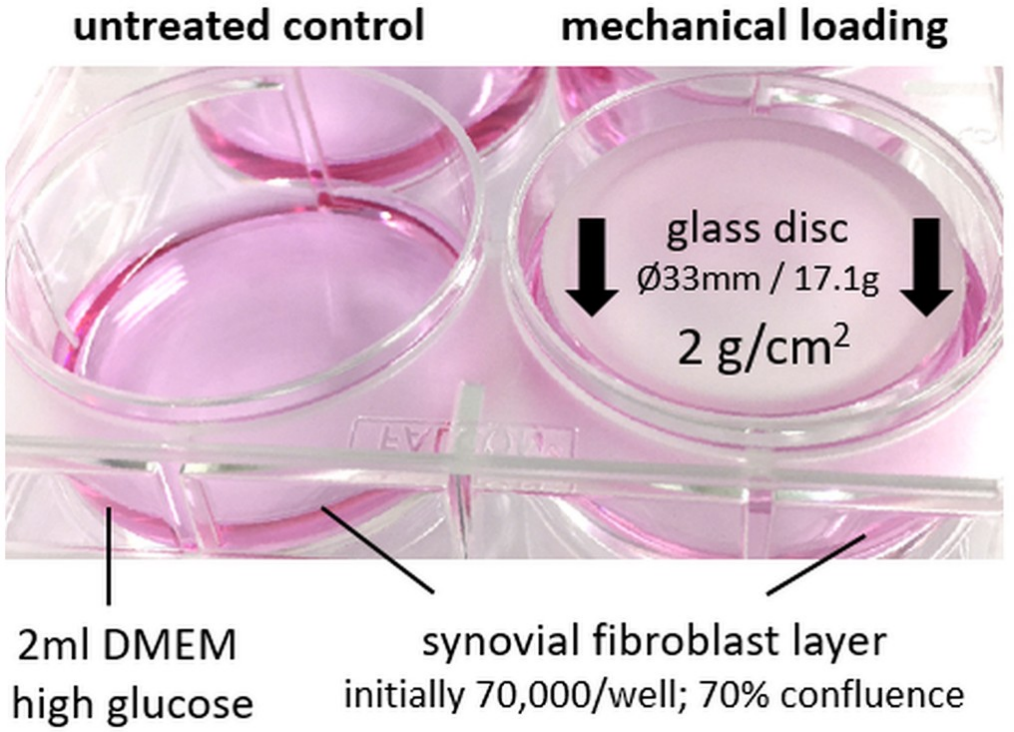
*In vitro* application of mechanical loading to synovial fibroblasts. Application of a sterile glass disc of defined size and weight to the cell layer, exerting a compressive force of 2g/cm^2^.

Approximately 70,000 synovial fibroblasts per well, either derived from a non-OA or an OA patient, were seeded on a 6-well plate and preincubated under cell culture conditions for 24 h. Afterwards incubation was continued for another 48 h with or without mechanical loading according to an established and published model for inducing compressive force on adherently growing fibroblasts [8,26,27]:

- 1st group: synovial fibroblasts from a healthy, non-OA patient (N-SF), incubated under cell culture conditions for a total of 72 h (n = 6);
- 2nd group: synovial fibroblasts from a healthy non-OA patient (N-SF) exposed to static compressive force (2 g/cm^2^ pressure, Fig 1) for 48 h after 24 h of preincubation (n = 6);
- 3rd group: synovial fibroblasts from an OA patient (OA-SF), incubated under cell culture conditions for a total of 72 h (n = 6);
- 4th group: synovial fibroblasts from an OA patient (OA-SF) exposed to static compressive force (2 g/cm^2^ pressure, Fig 1) for 48 h after 24 h of preincubation (n = 6).

### RNA isolation and purity determination

In order to remove residual cell culture media, synovial fibroblasts were washed two times with phosphate-buffered saline (PBS). RNA was isolated using peqGOLD TriFast^™^ (PEQLAB, 1 ml/well conforming with the manufacturer’s instructions [9,28,29]. The resulting RNA pellet was reconstituted in 25μl nuclease-free water (Carl-Roth) and immediately cooled on ice. Photometrical adsorption measurements at 280nm and 260nm revealed purity and quantity of the eluted RNA (NanoDrop, Implen). RNA integrity was determined using an Agilent 2100 Bioanalyzer (Agilent Technologies) based on the provided protocol of the manufacturer (S1 File). At least, one sample per group had to be excluded from further analysis due to poor or not measurable RNA integrity values (S2 File).

### Reverse transcription (cDNA synthesis)

For cDNA synthesis we transcribed 100 ng RNA per sample using a combination of 0.1 nmol random hexamer primer (Life Technologies), 0.1 nmol oligo-dT18 primer (Life Technologies) mixed with 1×M-MLV-buffer (Promega), 40 nmol dNTP mix (Carl-Roth), 40 U RNase inhibitor (Life Technologies) and M-MLV reverse transcriptase (Promega) and added nuclease-free H_2_O (Carl-Roth) to a final volume of 20 μl. We then incubated the samples at 37°C for 1 h and finally inactivated the reverse transcriptase at 95°C for 2 min. Experimental variation was reduced by simultaneous synthesis of cDNA for all samples.

### Real-time quantitative RT-PCR

The used oligonucleotides were designed based on the gene sequences achieved from the Nucleotide database NCBI (GeneBank, National Centre for Biotechnology Information) and validated for absence of secondary structures, self and cross dimers as well as primer efficiency and specificity, as already described [8] (Table 1). Eurofins MWG Operon LLC (Huntsville; High Purity Salt Free Purification HPSF^®^) was assigned for primer synthesis and purification.

**Table 1:** Information on tested genes, primer and target/amplicon for RT-qPCR.

Gene acronym	Gene name (Homo sapiens)	Gene function	Accession Number (NCBI GeneBank)	Chromosomal location (length)	5´-forward primer-3´ (length / T_m_ / %GC / max. ΔG Hairpin&Self-Dimer / Self-Comp. / Self-3’-Comp.)	5´-reverse primer-3´ (length / T_m_ / %GC / max. ΔG Hairpin&Self-Dimer / Self-Comp. / Self-3’-Comp.)	Primer Location (max. ΔG Cross-Dimer)	Amplicon (length, %GC, T_m_, SSAT)	Amplicon location (bp of Start/Stop)	Intron-flanking (length)	In silico qPCR specifity	Variants targeted
***EEF1A1***	eukaryotic translation elongation factor 1 alpha 1	enzymatic delivery of aminoacyl tRNAs to ribosome	NM_001402.5	6q14.1 (3528bp)	CCTGCCTCTCCAGGATGTCTAC (22bp / 64.0°C / 59.1% / -3.0 / 5 / 2)	GGAGCAAAGGTGACCACCATAC (22bp / 62.1°C / 54.6% / -3.2 / 6 / 2)	exon 5/6 (-2.9)	105bp, 52.4%,86.5°C, no SSAT	804/908	Yes (87bp)	Yes (BLAST/UCSC)	Yes
***GAPDH***	glyceraldehyde-3-phosphate dehydrogenase	enzyme in glycolysis and gluconeogenesis	NM_002046.5	12p13.31 (1421bp)	TGCCCTCAACGACCACTTTG (20bp / 59.4°C / 55.0% / -0.7 / 3 / 2)	CCACCACCCTGTTGCTGTAG (20bp / 61.4°C / 60.0% / 0.0 / 4 / 2)	exon 8/9 (-2.4)	74bp,50.0%,84.0°C, no SSAT	1091/1164	Yes (104bp)	Yes (BLAST/UCSC)	Yes
***POLR2A***	polymerase (RNA) II (DNA directed) polypeptide A, 220kDa	transcription of DNA into mRNA	NM_000937.4	17p13.1 (6738bp)	TCGCTTACTGTCTTCCTGTTGG (22bp / 60.3°C / 50.0% / 0.0 / 3 / 0)	TGTGTTGGCAGTCACCTTCC (20bp / 59.4°C / 55.0% / -1.3 / 3./ 3)	exon 21/22 (-2.5)	108bp, 53.7%,87.8°C, no SSAT	3798/3905	Yes (468bp)	Yes (BLAST/UCSC)	Yes
***PPIB***	peptidylprolyl isomerase A (cyclophilin B)	ER cyclosporine-binding protein	NM_000942.4	15q21-q22 (1045bp)	TTCCATCGTGTAATCAAGGACTTC (24bp / 59.3°C / 41.7% / -1.3 / 4 / 2)	GCTCACCGTAGATGCTCTTTC (21bp / 59.8°C / 52.4% / -0.7 / 4 / 0)	exon 3/4 (-2.1)	88bp, 53.4%,86.1°C, no SSAT	446/533	Yes (3194bp)	Yes (BLAST/UCSC)	Yes
***RNA18S***	18S ribosomal 5	ribosomal RNA, translation of mRNA in protein	NR_003286.2	22p12 (1869bp)	AACTGCGAATGGCTCATTAAATC (23bp / 57.1°C / 39.1% / -1.7 / 6 / 3)	GCCCGTCGGCATGTATTAG (19bp / 58.8°C / 57.9% / -2.4 / 5 / 1)	- (-2.4)	103bp, 46.6%,83.7°C, no SSAT	84/186	No (rRNA)	No (RNA45S5 also targeted)	-
***RPL22***	ribosomal protein L22	translation of mRNA in protein	NM_000983.3	1p36.31 (2099bp)	TGATTGCACCCACCCTGTAG (20bp / 59.4°C / 55.0% / -3.4 / 4 / 2)	GGTTCCCAGCTTTTCCGTTC (20bp / 59.4°C / 55.0% / -3.0 / 4 / 0)	exon 2/3 (-1.5)	98bp, 44.9%,83.8°C, no SSAT	115/212	Yes (4597bp)	Yes (BLAST/UCSC)	Yes
***RPLP0***	ribosomal protein, large, P0	translation of mRNA in protein	NM_001002.3	12q24.2 (1229bp)	GAAACTCTGCATTCTCGCTTCC (22bp / 60.3°C / 50.0% / -3.4 / 4 / 0)	GACTCGTTTGTACCCGTTGATG (22bp / 60.3°C / 50.0% / -2.0 / 4 / 0)	exon 6/7 (-1.8)	120bp, 50.8%,86.5°C, no SSAT	803/921	Yes (1091bp)	Yes (BLAST/UCSC)	Yes
***TBP***	TATA-box-binding protein	general transcription factor	NM_003194.4	6q27 (1921bp)	CGGCTGTTTAACTTCGCTTCC (21bp / 59.8°C / 52.4% / -0.8 / 5 / 0)	TGGGTTATCTTCACACGCCAAG (22bp / 60.3°C / 50.0% / -1.5 / 3 / 2)	exon 1/2 (-2.4)	86bp, 51.2%,85.6°C, no SSAT	79/164	Yes (2418bp)	Yes (BLAST/UCSC)	Yes
***YWHAZ***	tryptophan 5-monooxygenase activation protein, zeta	signal transduction,	NM_003406.3	8q23.1 (3003bp)	AGGAGATTACTACCGTTACTTGGC (24bp / 61.0°C / 46% / 0.0 / 4 / 2)	AGCTTCTTGGTATGCTTGTTGTG (23bp / 58.9°C / 43% / -3.0 / 4 / 0)	exon 8/9 (-2.2)	91bp, 47.3%,84.0°C, no SSAT	504/572	Yes (617bp)	Yes (BLAST/UCSC)	Yes
***COX2***	cyclooxygenase 2	prostaglandin synthesis	NM_000963.3	1q25.2–25.3 (4507bp)	GAGCAGGCAGATGAAATACCAGTC (24bp / 64.0°C / 50.0% / 0.0 / 2 / 2)	TGTCACCATAGAGTGCTTCCAAC (23bp / 63.1°C / 47.8% / -1.3 / 4 / 0)	exon 8/9 (-3.2)	131bp, 42.0%,82.9°C, no SSAT	1457/1587	Yes (486bp)	Yes (BLAST/UCSC)	Yes
***IL6***	interleukin 6	proinflammatory reaction	NM_000600.3	7p21 (1201bp)	TGGCAGAAAACAACCTGAACC (21bp / 61.7°C / 47.6% / -1.1 / 3 / 0)	CCTCAAACTCCAAAAGACCAGTG (23bp / 62.2°C / 47.8% / -0.8 / 3 / 3)	exon 2/3 (-1.5)	117bp, 43.6%,83.7°C, no SSAT	370/486	Yes (704bp)	Yes (BLAST/UCSC)	Yes
***COL1A2***	collagen, type I, alpha 2	encodes for collagen I type alpha 2	NM_000089.3	7q22.1 (5411bp)	AGAAACACGTCTGGCTAGGAG (21bp / 61.9°C / 52.4% / -3.3 / 4 / 2)	GCATGAAGGCAAGTTGGGTAG (21bp / 62.0°C / 52.4% / -2.3 / 5 / 0)	exon 50/51 (-0.7)	105bp, 44.8%,83.3°Cno SSAT	4139/4243	Yes (710bp)	Yes (BLAST/UCSC)	Yes
***P4HA1***	prolyl 4-hydroxylase, alpha polypeptide I	key enzyme in collagen synthesis	NM_000917.3	10q22.1 (2860bp)	GCTCTCTGGCTATGAAAATCCTG (23bp / 60.6°C / 47.8% / 0.0 / 2 / 2)	GTGCAAAGTCAAAATGGGGTTC (22bp / 58.4°C / 45.5% / -3.4 / 4 / 0)	exon 13/14 (-0.9)	146bp, 41.1%,82.2°C, no SSAT	1396/1541	Yes (13371bp)	Yes (BLAST/UCSC)	Yes

ER = endoplasmic reticulum; T_m_ = melting temperature of primer/specific qPCR product (amplicon); %GC = guanine/cytosine content; bp = base pairs; Comp. = Complementarity; SSAT = secondary structure at annealing temperature.

For each RT-qPCR reaction we used 7.5 μl SYBR^®^Green JumpStart^™^ Taq ReadyMix^™^ (Sigma-Aldrich), 10 pmol/μl of the respective forward and reverse primer and 1.5 μl of the diluted cDNA (1:10). RNase-free H_2_O (Carl-Roth) was added to a total volume of 15 μl. All cDNA samples were tested as three replicates per housekeeping gene and on the same 96 well PCR plate per biological replicate in 45 cycles (95°C for 5 min, per cycle 95°C for 10 s, 60°C for 8 s, 72°C for 8 s) to reduce possible inter-run variations on relative housekeeping gene stability assessment. Non-template controls and reverse transcription controls were additionally performed. For qPCR a Mastercycler^®^ ep realplex-S thermocycler (Eppendorf AG, Hamburg, Germany) was used in conjunction with 96-well PCR plates (Biozym Scientific) covered with BZO Seal Filmcover sheets (Biozym Scientific) [8,30,31].

### Assessment of reference gene stability

We calculated C_q_ values with the realplex software (version 2.2, Eppendorf AG) using the CalqPlex algorithm. The arithmetic mean of each C_q_ triplet per tested housekeeping gene and sample was used for analysis. The stability for each tested potential housekeeping gene was assessed by four different mathematical procedures: geNorm [12], NormFinder [13], BestKeeper [14] and the comparative ΔC_q_ method [16]. We performed stability calculations with the official corresponding Microsoft-Excel-based software applets according to the developers’ instructions. The comparative ΔC_q_ method was executed by manual calculations [16]. The application of the geNorm and NormFinder algorithms requires a transformation of native C_q_ data to the linear scale expression quantities Q = E^-(Cqmin-Cqsample)^ computing the qPCR efficiency (E) of each gene [8,9,16]. For evaluation, the tested housekeeping genes were listed based on their stability values (geNorm: M, NormFinder: ρ_ig_/σ_i_, deltaCT: mean SD of ΔC_q_; BestKeeper: Pearson’s r), determined by the chosen algorithms and set up. To assess the optimal number of houskeeping genes for solid RT-qPCR normalization, we used the geNorm [12] algorithm. A gene was considered superfluous for normalization, if the pairwise variation (V_n_/V_n+1_) of two pairs of housekeeping genes with one pair including an accessory gene was ≤ 0.15. By compilation of a bivariate correlation matrix (Pearson´s correlation coefficient r, two-sided, normality confirmed by Shapiro-Wilk tests and histogram evaluation) including the assessed stability values achieved by two respective algorithms, the variations between the tested mathematical procedures were ranked.

### Normalization of target gene expression

To assess the impact of housekeeping gene stability on relative expression of the target gene *prolyl-4-hydroxylase-alpha-1* (*P4HA1*), *collagen-1-alpha-2 (COL1A)*, *cylclooxygenase-2 (COX2)* and *interleukin-6 (IL6)* we determined the relative gene expression as 2^-ΔCq^ [32] with ΔC_q_ = C_q_ (target gene)–C_q_ (housekeeping gene), divided by the respective arithmetic 2^-ΔCq^ mean of the untreated synovial fibroblast controls derived from a healthy subject with their relative gene expression normalized as 1. Using the software application SPSS^®^ Statistics 24 (IBM^®^, Armonk, NY, USA), data were tested for normal distribution (Shapiro-Wilk test) and homogeneity of variance (Levene’s test). Experimental groups were compared by Welch-corrected one-way ANOVAs. We used Games–Howell post hoc tests for pairwise comparisons. All differences were considered statistically significant at p≤0.05. Descriptive statistics are given as arithmetic mean ± standard deviation.

## Results

### Quality and integrity of RNA samples

The mean concentration of harvested RNA (n = 20) was assessed by its optical density (260nm) as 25.5 ng/μl (standard deviation SD 10.8 / Min. 12.1 / Max. 46.4) with a mean OD_260nm/280nm_ ratio of 1.91 (SD 0.14 / Min. 1.82 / Max. 2.21) indicating a negligible contamination with protein (S1 Table). The RNA integrity number (RIN) algorithm allocates a RIN number score from 1 to 10 with a value of 10 representing completely intact RNA and a value of 1 degraded RNA [33]. Three samples displayed poor RIN values and one sample concentration was too low for RIN measurement. These samples were excluded from housekeeping gene analysis. For the other samples RIN values ranged from 9.3 to 9.8 (mean 9.2, SD 0.4), indicating negligible RNA degradation (S1 File). We also confirmed integrity of total RNA by assessing the ratio of 28S/18S ribosomal RNA, which ranged from 1.1 to 2.4 (S1 File). The negative controls (reverse transcription negative control, negative NTC reactions) did not show the presence of interfering genomic DNA and contamination, as the observed C_q_ values were substantially higher than those of of wells containing samples or reverse transcriptase (S2 Table).

### Primer specificity and C_q_ expression levels

**Fig 2:**
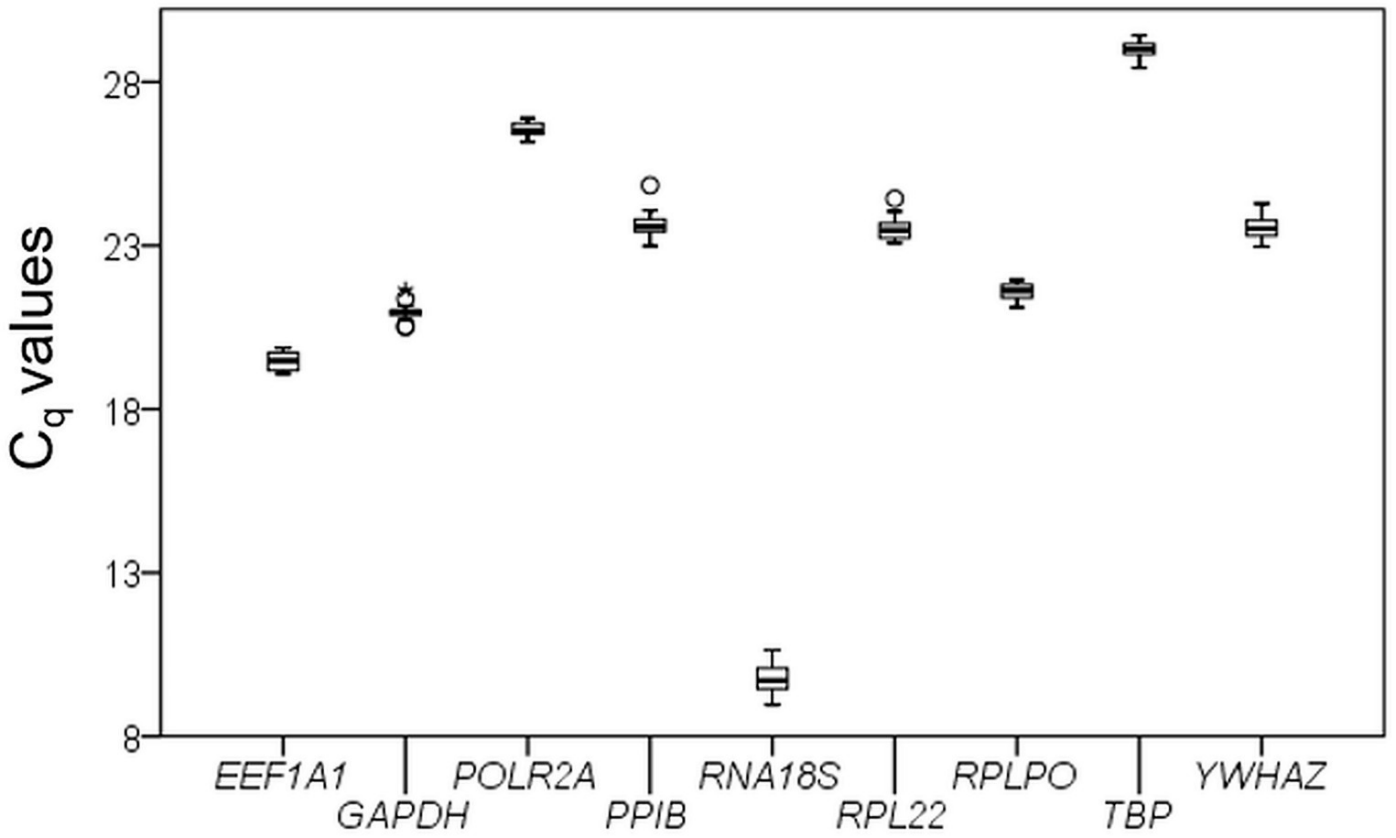
Expression levels of tested housekeeping genes in the experimental set-up. Values illustrate mean C_q_ (quantification cycle; n = 20). Gene names are listed in Table 1. Boxplots illustrate median, interquartile range as box and data range by whiskers. Circles show outliers and asterisms extreme values.

We confirmed primer specificity by agarose gel electrophoresis and melting curve analysis (S2 File). The range of observed C_q_ values of the tested genes was between 9.0 to 29.4 cycles (Fig 2, S3 Table). *RNA18S* showed the lowest values and *TBP* the highest.

### Refinement of housekeeping gene number for normalization

**Fig 3:**
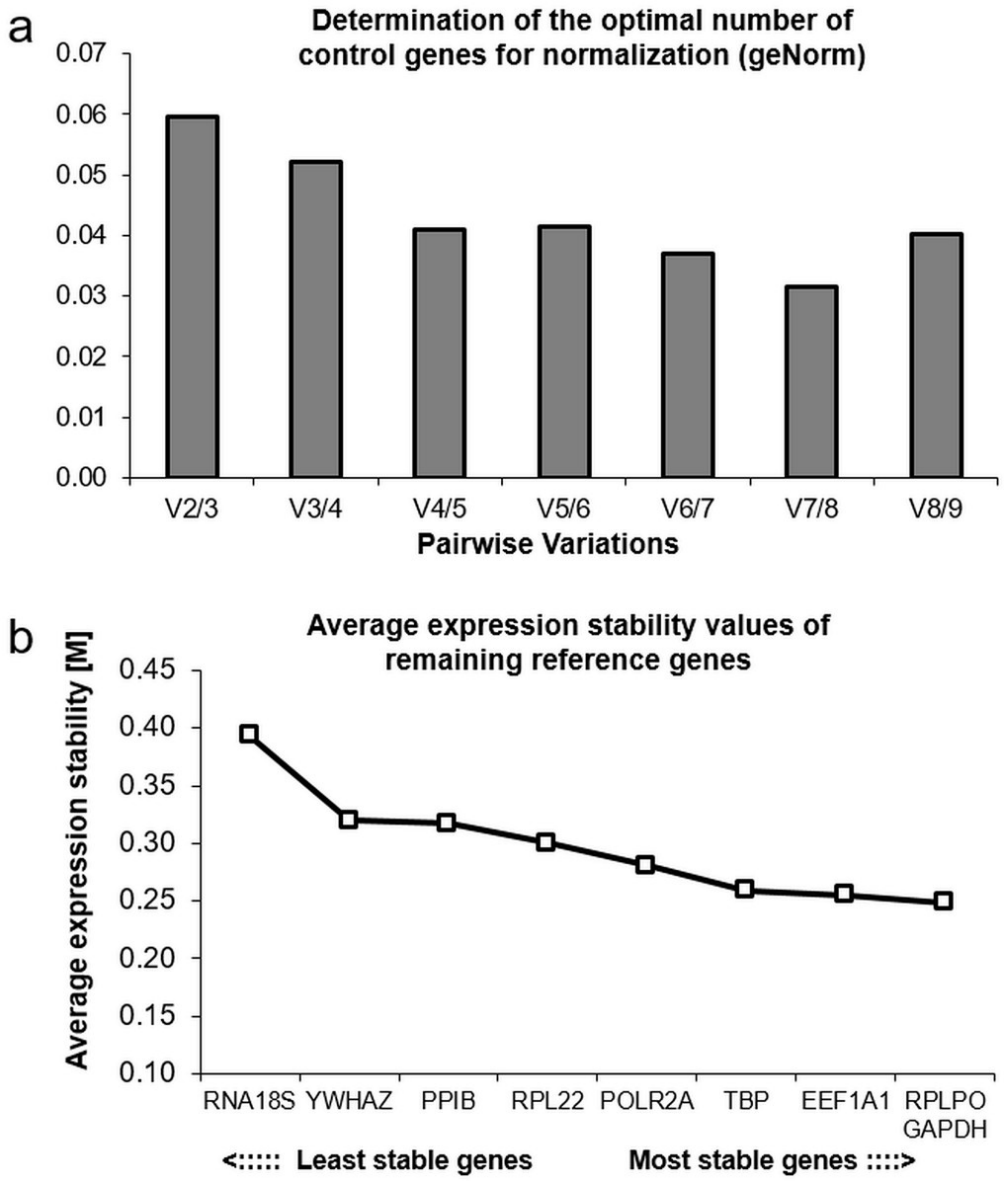
Analysis of expression stability of the tested housekeeping genes with geNorm. (a) Ideal number of housekeeping genes for gene expression studies on synovial fibroblasts. V = variation (b) Average values of expression stability derived by successive exclusion of the most instable performed housekeeping gene across all specimens and experimental conditions (n = 20). Decreasing M values indicate increasing gene expression stability. Consult Table 1 for gene names.

For studies with synovial fibroblasts, two housekeeping genes in RT-qPCR in the tested experimental conditions were adequate for normalization according to the geNorm algorithm using their geometric mean (Fig 3a).

### Relative performance as housekeeping gene

By using the geNorm algorithm, we found *RPLP0* and *GAPDH* to be the most stably expressed housekeeping genes overall (Table 2, Fig 3b). Similarly, comparing N-SF and OA-SF fibroblasts without mechanical loading with the geNorm algorithm, we determined *RPLP0*, *GAPDH* and *PPIB* to have the lowest expression variation (Table 2). Additionally, we also analysed untreated N-SF and compressive-force-treated N-SF with this algorithm and revealed *EEF1A1* and *RPLP0* to be the most stably expressed housekeeping genes under these conditions (Table 2).

**Table 2:** Housekeeping gene stability ranking for human synovial fibroblast experiments with compressive force application (compressive force vs. untreated control), experiments on osteoarthritis (osteoarthritis vs. normal fibroblasts) and pooled/overall experimental conditions. Calculations based on the algorithms geNorm, NormFinder, comparative ΔC_q_ and BestKeeper. A higher rank indicates reduced expression stability.

Rank	Total (of 4 methods)		geNorm		NormFinder			comparative deltaC_q_		BestKeeper			
	Ranking order	Rank sum	Ranking order	Stability value (M)	Ranking order	Stability value (ρ_ig_/σ_i_)	Standard error	Ranking order	Stability value (mean SD of mean ΔC_q_)	Ranking order	Stability value (r)	SD (+/- C_q_)	CV (% C_q_)
**Synovial fibroblasts pooled/overall** (experiments on mechanical loading and osteoarthritis, n = 20)													
1.)	***RPLP0***	**6**	***RPLP0***	**0.242**	***RPLP0***	**0.082**	**0.019**	***RPLP0***	**0.255**	***EEF1A1***	**0.884**	**0.234**	**1.203**
2.)	***EEF1A1***	**8**	***GAPDH***	**0.254**	***EEF1A1***	**0.097**	**0.021**	***EEF1A1***	**0.264**	***RNA18S***	**0.871**	**0.385**	**3.958**
3.)	*GAPDH*	14	*EEF1A1*	0.255	*TBP*	0.103	0.022	*GAPDH*	0.267	*RPLP0*	0.862	0.211	0.975
4.)	*TBP*	17	*TBP*	0.259	*GAPDH*	0.106	0.022	*TBP*	0.274	*RPL22*	0.824	0.282	1.200
5.)	*RPL22*	22	*POLR2A*	0.281	*POLR2A*	0.133	0.025	*POLR2A*	0.295	*TBP*	0.796	0.207	0.713
6.)	*POLR2A*	24	*RPL22*	0.300	*RPL22*	0.149	0.027	*RPL22*	0.312	*GAPDH*	0.741	0.171	0.816
7.)	*PPIB*	28	*PPIB*	0.317	*PPIB*	0.173	0.031	*PPIB*	0.337	*PPIB*	0.709	0.269	1.139
8.)	*RNA18S*	29	*YWHAZ*	0.320	*YWHAZ*	0.175	0.031	*YWHAZ*	0.337	*YWHAZ*	0.662	0.297	1.261
9.)	*PPIB*	32	*RNA18S*	0.394	*RNA18S*	0.244	0.041	*RNA18S*	0.395	*POLR2A*	0.619	0.182	0.686
**N-SF vs. OA-SF** (experiments on osteoarthritis, n = 10)													
1.)	***RPLP0***	**6**	***RPLP0***	**0.202**	***RPLP0***	**0.064**	**0.023**	***RPLP0***	**0.213**	***RNA18S***	**0.894**	**0.319**	**3.228**
2.)	***TBP***	**12**	***GAPDH***	**0.214**	***PPIB***	**0.081**	**0.025**	***PPIB***	**0.225**	***TBP***	**0.843**	**0.195**	**0.671**
3.)	***PPIB***	**12**	***PPIB***	**0.214**	*GAPDH*	0.085	0.026	*TBP*	0.241	*RPLP0*	0.841	0.188	0.869
4.)	*GAPDH*	20	*TBP*	0.226	*TBP*	0.093	0.027	*POLR2A*	0.252	*EEF1A1*	0.752	0.203	1.042
5.)	*EEF1A1*	21	*POLR2A*	0.239	*POLR2A*	0.116	0.031	*EEF1A1*	0.254	*RPL22*	0.721	0.220	0.936
6.)	*POLR2A*	21	*EEF1A1*	0.249	*RPL22*	0.128	0.034	*RPL22*	0.263	*PPIB*	0.692	0.134	0.571
7.)	*RPL22*	23	*RPL22*	0.254	*EEF1A1*	0.130	0.034	*YWHAZ*	0.332	*GAPDH*	0.636	0.123	0.586
8.)	*RNA18S*	25	*RNA18S*	0.303	*RNA18S*	0.178	0.044	*GAPDH*	0.598	*POLR2A*	0.549	0.141	0.535
9.)	*YWHAZ*	33	*YWHAZ5*	0.314	*YWHAZ*	0.193	0.048	*RNA18S*	0.679	*YWHAZ*	0.478	0.208	1.192
**N-SF untreated vs. compressive force** (experiments on pressure application, n = 10)													
1.)	***EEF1A1***	**4**	***EEF1A1***	**0.217**	***EEF1A1***	**0.064**	**0.025**	***EEF1A1***	**0.224**	***EEF1A1***	**0.936**	**0.225**	**1.154**
2.)	***RPLP0***	**9**	***RPLP0***	**0.221**	***RPLP0***	**0.078**	**0.026**	***RPLP0***	**0.234**	***TBP***	**0.913**	**0.191**	**0.658**
3.)	*TBP*	11	*TBP*	0.225	*TBP*	0.080	0.027	*TBP*	0.238	*RPLP0*	0.897	0.229	1.057
4.)	*RPL22*	16	*RPL22*	0.236	*RPL22*	0.100	0.030	*RPL22*	0.246	*RPL22*	0.873	0.224	0.950
5.)	*GAPDH*	21	*GAPDH*	0.246	*GAPDH*	0.109	0.031	*GAPDH*	0.259	*RNA18S*	0.803	0.319	3.247
6.)	*YWHAZ*	26	*YWHAZ*	0.253	*YWHAZ*	0.120	0.033	*YWHAZ*	0.266	*GAPDH*	0.774	0.200	0.951
7.)	*POLR2A*	30	*POLR2A*	0.278	*POLR2A*	0.145	0.038	*POLR2A*	0.295	*PPIB*	0.757	0.313	1.322
8.)	*RNA18S*	31	*PPIB*	0.354	*PPIB*	0.219	0.054	*RNA18S*	0.362	*YWHAZ*	0.639	0.167	0.699
9.)	*PPIB*	32	*RNA18S*	0.358	*RNA18S*	0.225	0.055	*PPIB*	0.380	*POLR2A*	0.597	0.204	0.768

C_q_ = quantification cycle; SD = standard deviation; CV = coefficient of variation; r = Pearson’s correlation coefficient.

The NormFinder algorithm identified *RPLP0* and *EEF1A1* as most stable genes overall (Table 2). NormFinder confirmed the geNorm findings for N-SF and OA-SF fibroblasts without compressive force application and for untreated and loaded N-SF (Table 2), as this algorithm also revealed *RPLP0* and *PPIB* or *EEF1A1* and *RPLP0* as most stably expressed housekeeping genes.

The comparative ΔC_q_ method [16] was in line with NormFinder results (Table 2) regarding the combined conditions, as it also defined *RPLP0* and *EEF1A1* to be the most stably expressed genes. For the other tested conditions, the comparative ΔC_q_ method confirmed the findings of NormFinder and geNorm algorithms (Table 2).

The BestKeeper algorithm [14] was also in accordance with NormFinder and the comparative ΔC_q_ method suggesting *EEF1A1* as the best housekeeping gene overall (Table 2). For experiments regarding N-SF and OA-SF experiments BestKeeper defined *RNA18S* and *TBP* as reliable housekeeping genes (Table 2). For N-SF treated with or without compressive force BestKeeper proposed *EEF1A1* and *TBP* as suitable housekeeping genes (Table 2).

### Conformity of used algorithms for housekeeping gene stability analysis

**Fig 4:**
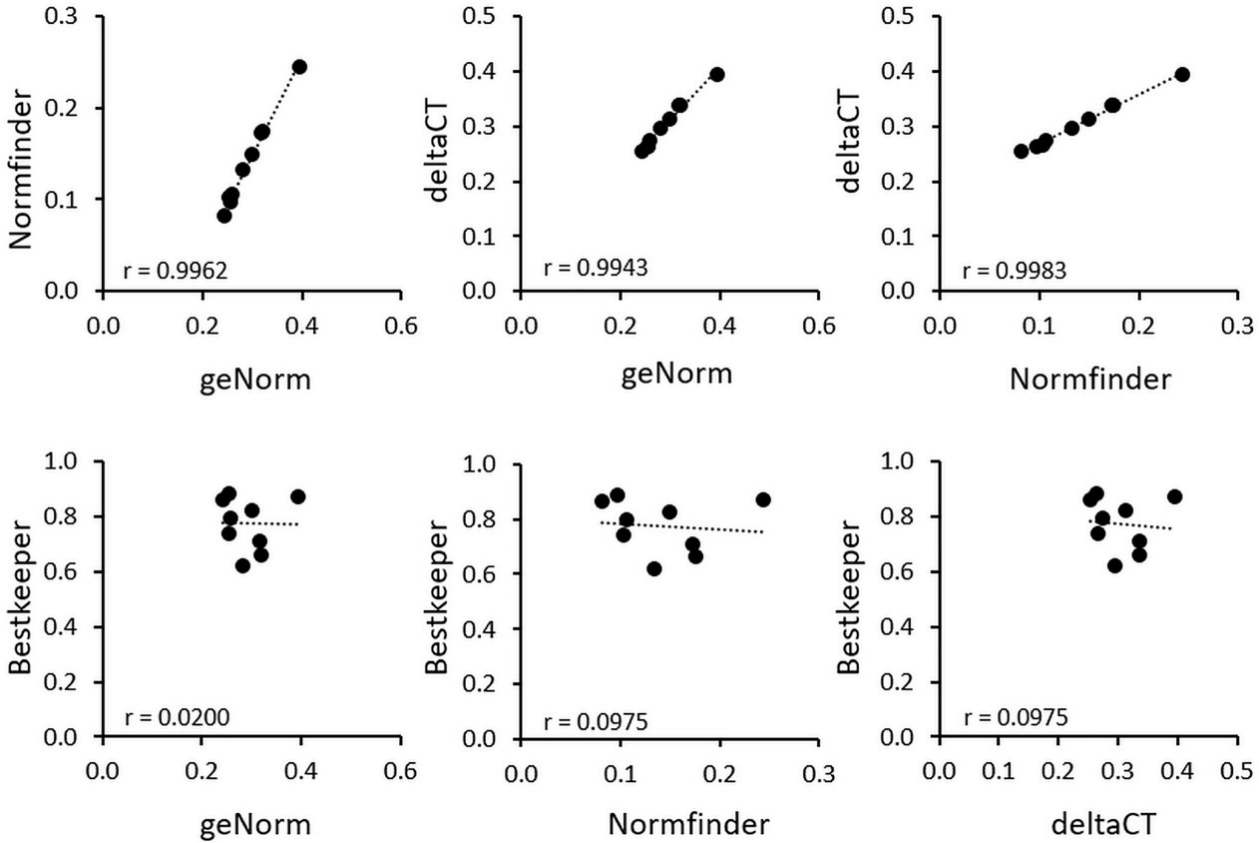
Correlation analysis of values for stability of the conducted algorithms for housekeeping gene assessment (geNorm, NormFinder, BestKeeper, comparative ΔC_q_). Bivariate correlations are displayed by scatterplots of the total stability values of the tested housekeeping genes, calculated by two algorithms. Correlation plots include linear regression lines.

We performed bivariate correlations of gene rankings between the used algorithms to assess their conformity (Fig 4, n = 20). geNorm, NormFinder and the comparative ΔC_q_ method display significant correlations of the ranking of the tested housekeeping genes. Only BestKeeper algorithm showed discrepancies, as it did not correlate with the other algorithms used (Fig 4).

### Effect of housekeeping gene stability on relative target gene expression

**Fig 5:**
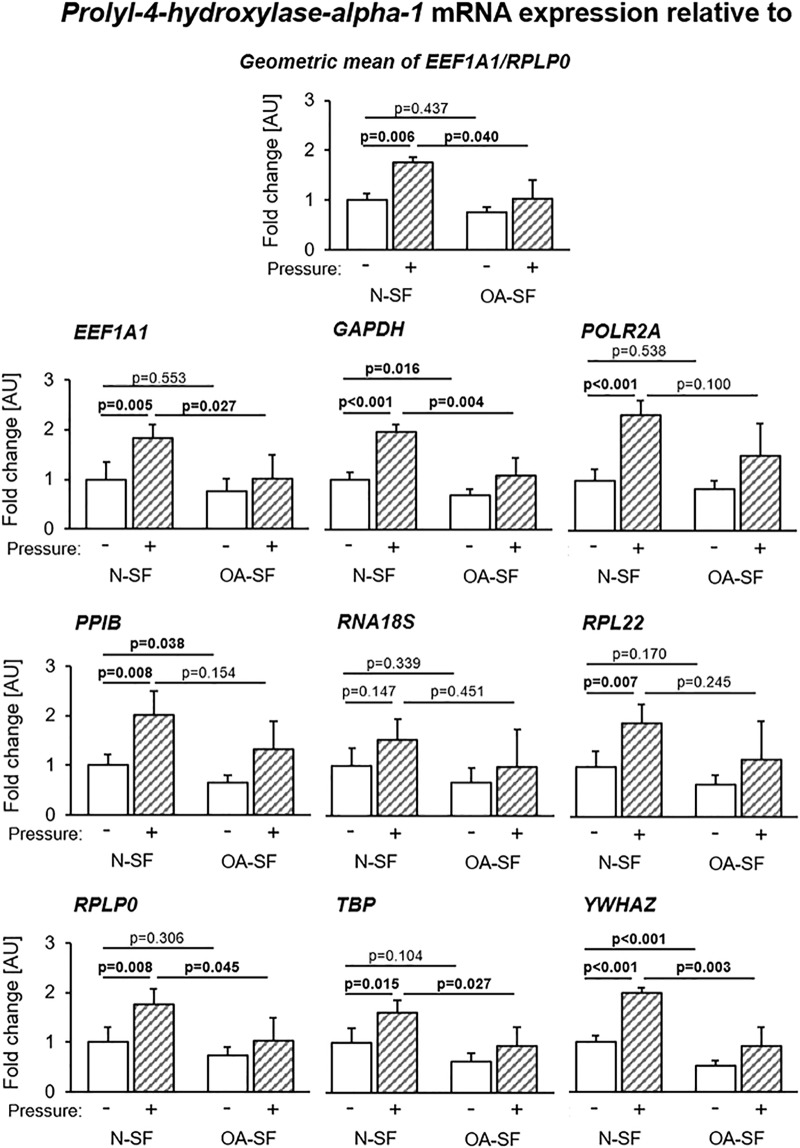
Influence of the choice of housekeeping gene used for normalization and its stability on the fold-change expression of *P4HA1* in N-SF and OA-SF without and with additional static pressure application. Distinct differences in relative gene expression are evident with significance of pairwise comparisons varying across the individual housekeeping genes used for normalization (n = 6 per group). Statistics: Welch-corrected ANOVA with Games-Howell post-hoc tests.

To assess the importance of the correct choice of housekeeping genes for experiments on N-SF and OA-SF with and without mechanical loading, we calculated relative gene expression of the target gene *prolyl-4-hydroxylase-alpha-1* (*P4HA1*, Fig 5), *collagen-1-alpha-2* (*COL1A2*), *cylcooxygenase-2* (*COX2*) and *interleukin-6* (*IL-6*; all in S3 File) using the candidate housekeeping genes tested in this study, which differ in expression stability. *P4HA1* is responsible for the proper three-dimensional folding of newly synthesized procollagen chains. The expression levels of *P4HA1* varied in a wide range depending on the housekeeping gene used for normalization (Fig 5). We observed significant pressure effects in N-SF with *EEF1A1*, *GAPDH*, *POLR2A*, *RNA18S*, *RPL22*, *RPLP0* and *YWHAZ* used for normalization, but using *PPIB* as housekeeping gene, we determined no significant induction of *P4HA1* (Fig 5). Differences between N-SF and OA-SF under physiological conditions seemed to be significant with *GAPDH*, *PPIB* and *YWHAZ*. With all other tested housekeeping genes there were no significant differences in *P4HA1* expression between N-SF and OA-SF detectable (Fig 5). Variations between N-SF and OA-SF after compressive force treatment only appeared significant with *EEF1A1*, *GAPDH*, *RPLP0*, *TBP* and *YWHAZ* as housekeeping genes (Fig 5), but not with *POLR2A*, *PPIB*, *RNA18S* or *RPL22*.

Furthermore, we calculated the statistical differences between results of target gene analysis for *P4HA1*, *COL1A2*, *COX-2* and *IL-6* normalized to the least stable housekeeping gene PPIB and the geometric mean of *EEF1A1/RPLP0* as most stable housekeeping genes using an unpaired t test for the different conditions and groups (intragroup comparisons, S4 Table). We determined a significant difference only for *IL-6* target gene expression in one experimental condition. Nonetheless, there are differences in significance levels due to normalization with different reference genes (Fig 5, S3 File).

## Discussion

We identified *RPLP0*, *EEF1A1*, *TBP* and *PPIB* to be reliable housekeeping genes for normalization of target gene expression in RT-qPCR studies on human synovial fibroblasts derived from non-OA or OA patients by analysing the expression stability of at least nine potentially suitable housekeeping genes with four different mathematical algorithms. In general *RPLP0* and *EEF1A1* proved to be most stable throughout all tested experimental conditions and algorithms. As required by the MIQE guidelines, these genes have different functions in cell metabolism, which indicates that they are not co-regulated and may be used in conjunction for normalization of gene expression [10]. *RPLP0* is involved in protein synthesis, as this gene encodes for one large 60S acidic ribosomal protein subunit [34]. *EEF1A1* encodes for an isoform of the alpha subunit of the elongation factor-1 complex [35], which acts as GTPase and actin-bundling protein [36]. *RPLP0*, *TBP* and *PPIB* seem to be ideal for studies focusing on differences between non-OA and OA synovial fibroblasts. In contrast, *PPIB* encodes for a protein-binding cyclosporine in the endoplasmic reticulum, which is important in collagen type I folding [37]. *PPIB* is also associated with pathological conditions potentially affecting osteoarthritis, such as osteogenesis imperfecta [38]. *TBP* encodes for a TATA-box-binding protein, which is involved in transcription processes by the regulation of the RNA polymerase I [39]. A set of two internal control genes were determined to be adequate for reliable reference normalization using their geometric mean.

We confirmed high intraassay reliability and precision of the obtained data [40], based on the satisfactory quality of the obtained RNA samples and RT-qPCR analysis. We confirmed protein-free and qualitative adequate RNA by assessment of the purity and integrity of total isolated RNA, as protein contamination can inhibit cDNA synthesize and qPCR reaction and therefore lead to biased C_q_ values [41]. We also confirmed primer specificity *in silico* and *in vitro*.

Various studies on other tissues and experimental setups exist, which have determined stable housekeeping genes in the used conditions and tissues [42–44]. But most of them used only two algorithms for housekeeping gene stability assessment [11,45]. In this study we used four mathematical procedures and evaluated their conformity to assess, whether a combined usage of these algorithms adds reliability in housekeeping gene stability calculations. NormFinder, geNorm and comparative ΔC_q_ algorithms correlated to a a high degree with each other, which was mirrored by similar gene stability rankings. The BestKeeper algorithm on the other hand differed in its assessment from the other procedures. Originally, it was designed to assess general suitability of a housekeeping gene for RT-qPCR in a consecutive two-step assessment based on mean C_q_ standard deviation and correlation analysis and not to compare possible housekeeping genes. The other algorithms like geNorm or the comparative ΔC_q_ method implement either pairwise comparisons of housekeeping genes with linear quantities or apply an approach of linear quantity models, as it can be seen in the NormFinder algorithm [13].

To assess the impact of choosing appropriate housekeeping genes for normalization, we calculated *P4HA1* gene expression normalized to the different candidate housekeeping genes. We observed distinct differences in the significance levels attributable to the relative stability of the respective housekeeping gene used for normalization. These results confirm the importance of housekeeping gene validation and of proper selection of stably expressed housekeeping genes in experiments on N-SF and OA-SF synovial fibroblasts.

## Conclusions

We identified *RPLP0*, *EEF1A1*, *TBP* and *PPIB* to be reliable housekeeping genes for normalization of target gene expression in RT-qPCR studies on human synovial fibroblasts derived from non-OA or OA patients by analysing the expression stability of at least nine potentially suitable housekeeping genes with four different mathematical procedures. *RPLP0* and *EEF1A1* proved to be the most stably expressed housekeeping genes regarding studies on synovial fibroblasts focusing on experimental compressive force loading, whereas *RPLP0*, *TBP* and *PPIB* seem to be ideal for studies focusing on differences between non-OA and OA synovial fibroblasts. For accurate normalization, a set of two housekeeping genes was determined as sufficient, when using their geometric mean.

## Supporting information

S1 Table Yield (quantity) and quality of extracted total RNA per biological replicate (well). (DOCX)

S2 Table MIQE checklist for authors, reviewers and editors. (DOCX)

S3 Table Raw Cq values of RT-qPCR for the two experimental groups and nine potentially suitable housekeeping genes. (DOCX)

S4 Table Statistical differences given as p values in each experimental group (intragroup comparisons) between results of target gene analysis for *P4HA1*, *COL1A2*, *COX-2* and *IL-6* normalized to a single reference gene and results normalized to the geometric mean of *EEF1A1/RPLP0*. *Statistics*: unpaired t-test using GraphPad Prism version 8.0.
(DOCX)

S1 File RNA integrity analysis. (PDF)

S2 File Primer specificity evaluation via (a) agarose gel and (b) melting curve analysis of all used samples (left) and a single sample (right). (PDF)

S3 File Influence of the choice of housekeeping gene used for normalization on the fold-change expression of *COL1A2*, *COX2* and *IL6* in N-SF and OA-SF without and with additional static pressure application. (PDF)
