# Supplementary material for: Housekeeping gene validation for RT-qPCR studies on synovial fibroblasts derived from healthy and osteoarthritic patients with focus on mechanical loading
Source: PLoS One. 2019 Dec 6;14(12):e0225790. doi: 10.1371/journal.pone.0225790 (PMC6897414; doi:10.1371/journal.pone.0225790)
Supplement: S1 Table — (DOCX) [file pone.0225790.s001.docx]

**S1 Table. Yield (quantity) and quality of extracted total RNA per biological replicate (well).**

| **Experiment** | **Sample ID** | **RNA concentration [ng/µl]** | **A260** | **A280** | **A260/A280** |
| --- | --- | --- | --- | --- | --- |
| hSF02 | Sample 1 | 32.120 | 0.810 | 0.370 | 2.212 |
| hSF02* | Sample 2* | 37.360 | 0.936 | 0.477 | 1.966 |
| hSF02 | Sample 3 | 32.320 | 0.806 | 0.408 | 1.971 |
| hSF02* | Sample 4* | 18.120 | 0.461 | 0.250 | 1.872 |
| hSF02 | Sample 5 | 19.080 | 0.479 | 0.259 | 1.856 |
| hSF02 | Sample 6 | 14.160 | 0.344 | 0.180 | 1.863 |
| hSF02 | Sample 7 | 34.040 | 0.846 | 0.437 | 1.925 |
| hSF02 | Sample 8 | 29.480 | 0.724 | 0.362 | 1.965 |
| hSF02 | Sample 9 | 30.280 | 0.740 | 0.377 | 1.921 |
| hSF02* | Sample 10* | 8.2800 | 0.180 | 0.120 | 1.408 |
| hSF02 | Sample 11 | 15.360 | 0.369 | 0.203 | 1.761 |
| hSF02 | Sample 12 | 13.720 | 0.321 | 0.166 | 1.824 |
| hSF06 | Sample 1 | 35.800 | 0.904 | 0.454 | 2.011 |
| hSF06 | Sample 2 | 35.200 | 0.886 | 0.444 | 2.009 |
| hSF06 | Sample 3 | 37.360 | 0.941 | 0.485 | 1.954 |
| hSF06 | Sample 4 | 24.400 | 0.615 | 0.330 | 1.877 |
| hSF06 | Sample 5 | 16.480 | 0.418 | 0.219 | 1.934 |
| hSF06 | Sample 6 | 12.080 | 0.302 | 0.154 | 1.961 |
| hSF06 | Sample 7 | 36.080 | 0.900 | 0.468 | 1.919 |
| hSF06 | Sample 8 | 46.400 | 1.162 | 0.591 | 1.969 |
| hSF06 | Sample 9 | 36.080 | 0.899 | 0.450 | 1.991 |
| hSF06 | Sample 10 | 14.520 | 0.361 | 0.192 | 1.871 |
| hSF06* | Sample 11* | 20.880 | 0.520 | 0.276 | 1.878 |
| hSF06 | Sample 12 | 12.920 | 0.316 | 0.166 | 1.867 |

A = absorbance = optical density (OD) at 260nm and 280nm; A260/A280 = absorbance ratio. Factor = ng/µl total RNA per 1 unit OD_260nm_. *: Samples were excluded from ranking due to insufficient RIN value.
