# Supplementary material for: Housekeeping gene validation for RT-qPCR studies on synovial fibroblasts derived from healthy and osteoarthritic patients with focus on mechanical loading
Source: PLoS One. 2019 Dec 6;14(12):e0225790. doi: 10.1371/journal.pone.0225790 (PMC6897414; doi:10.1371/journal.pone.0225790)
Supplement: S2 Table — (DOCX) [file pone.0225790.s002.docx]

**S2 Table. MIQE checklist for authors, reviewers and editors.** E = essential information; D = desirable information.

| **Item to check** | **Importance** | **Description how item was addressed in study / article** |
| --- | --- | --- |
| **Experimental design** | | |
| Definition of experimental and control groups | E | 1^st^ group: untreated normal synovial fibroblasts (N-SF, physiological conditions); 2^nd^ group: N-SF with compressive force application. 3^rd^ group: untreated synovial fibroblasts derived from an osteoarthitis patient (OA-SF, physiological conditions); 4^th^ group: OA-SF with pressure application. For details see materials and methods and Figure 1. |
| Number within each group | E | n = 6 |
| Assay carried out by the core or investigator´s laboratory? | D | All assays were carried out in investigators’ laboratory. |
| Acknowledgment of authors´ contributions | D | C.K. and A.S. conceived the study design. C.K. designed and validated the used primer pairs. U.N., S.G., P.P. and D.M. contributed to discussion and study design. U.N. and A.S. conducted the experiments as well as analysed the results. U.N., A.S. and C.K. wrote the manuscript and created the figures, tables and the supplementary material. All authors reviewed the manuscript. |
| **Sample** | | |
| Description | E | Synovial fibroblasts from a healthy, non-OA patient were obtained from BioIVT (PCD-90-0645). Synovial fibroblasts from an OA patient were kindly provided by the Department of Orthopaedics, University of Regensburg (SG). |
| Volume/mass of sample processed | D | 70,000 fibroblasts were seeded per well / biological replicate for the experiments. |
| Microdissection or macrodissection | E | Microdissection |
| Processing procedure | E | Synovial fibroblasts were cultivated under normal cell culture conditions (37°C, 5% CO_2_, water-saturated) in full media (DMEM, high glucose) until the 6^th^ passage. |
| If frozen, how and how quickly? | E | Until use synovial fibroblasts were frozen in liquid nitrogen (90% FBS, 10% DMSO, freezing 1°C/minute in cryo-box with isopropanol). |
| If fixed, with what and how quickly? | E | Not fixed. |
| Sample storage conditions and duration | E | Samples were directly isolated, cultivated under cell culture conditions and stored in liquid nitrogen until use. |
| **Nucleid acid extraction** | | |
| Procedure and/or instrumentation | E | After washing fibroblasts twice with sterile phosphate-buffered saline, total RNA from synovial fibroblasts was extracted by applying peqGOLD TriFast™ and further processing according to the manufacturer’s instructions. We eluted the resulting RNA pellet in nuclease-free water (25µl) with immediate ice-cooling. |
| Name of kit and details of any modifications | E | peqGOLD TriFast™ (1 ml / well, PEQLAB Biotechnology GmbH, Erlangen, Germany). We followed the manufacturer´s protocol exactly. |
| Source of additional reagents used | D | Chloroform (EMSURE^®^, 1.02445.1000; Merck KG, Darmstadt, Germany), 2-Propanol (20842.330, VWR International GmbH, Darmstadt, Germany), Ethanol (32205, Sigma-Aldrich, Munich, Germany); RNase-free water (T143, Bioscience-Grade, Carl Roth GmbH & Co. KG, Karlsruhe, Germany) |
| Details of DNase or RNase treatment | E | 1 µg of RNA was treated with 40 U of RNase inhibitor (EO0381, Life Technologies) in a 22 µl final volume for cDNA synthesis. No DNAse treatment was performed. |
| Contamination assessment (DNA or RNA) | E | We tested a no-template-control (NTC) without cDNA and a -RT control (cDNA synthesis without enzyme reverse transcriptase added) for each primer pair and qPCR run on the same plate to exclude possible bias by primer dimers, contaminating or genomic DNA. |
| Nucleic acid quantification | E | RNA concentration was determined by measuring the absorbance at 260 nm UV light with 1 OD_260nm_ equalling 40 ng/µl total RNA. OD = optical density |
| Instrument and method | E | NanoDrop (Implen, Munich) |
| Purity (A260/A280) | D | We determined RNA purity by measuring the absorbance ratio OD_260nm/280nm_. An OD_260nm/280nm_ ratio of >1.8 was considered protein-free RNA (Supplementary Table 2). |
| Yield | D | RNA yield was calculated as the amount of RNA obtained (µg) per well. **Mean yield:** 25.5 ng/µl x 20 µl/sample = 510.0 ng/sample; **Min./Max. yield:** 8.3 / 46.4 ng/µl x 20 µl/sample = 166 / 928 ng/sample (Supplementary Table 2). |
| RNA integrity: method/instrument | E | RNA integrity was determined with an Agilent 2100 Bioanalyzer (Agilent Technologies Inc. Santa Clara, CA, USA) according to the manufacturer’s protocol (Supplementary Data 1). |
| RIN/RQI or C_q_ of 3´ and 5´ transcripts | E | Mean RIN values were 8.71 ± 1.5 SD with excluded samples and 9.2 ± 0.4 SD without excluded samples (Supplementary Data 1). |
| Electrophoresis traces | D | Electrophoresis traces were determined with an Agilent 2100 Bioanalyzer (Agilent Technologies Inc. Santa Clara, CA, USA) according to the manufacturer’s protocol (Supplementary Data 1). |
| **Reverse transcription** | | |
| Complete reaction conditions | E | For cDNA synthesis, we transcribed a standardized quantity of 100 ng RNA per sample using 1 µl random hexamer primer (0.1 nmol, Life Technologies), 1 µl oligo-dT18 primer (0.1 nmol, Life Technologies), 4 µl 5×M-MLV-buffer (Promega), 1 µl dNTP mix (40 nmol, dNTP, Carl-Roth), 1 µl RNase inhibitor (40 U, Life Technologies), 1 µl reverse transcriptase (200 U, Promega) and added nuclease-free H_2_O (Carl-Roth) to a final volume of 20 µl. We then incubated the samples at 37°C for 60 min and heat-inactivated the reverse transcriptase at 95°C for 2 min. To minimize experimental variations, synthesis of cDNA was performed concurrently for all samples. cDNA was stored at −20°C until use. |
| Amount of RNA and reaction volume | E | **Amount of RNA:** 100 ng; **Reaction volume:** 20 µl |
| Priming oligonucleotide (if using GSP) and concentration | E | 0.1 nmol random hexamer primer; 0.1 nmol oligo-dT18 primer |
| Reverse transcriptase and concentration | E | Reverse transcriptase (200 U, 1 µl, Promega) in a final concentration of 9.1 U/µl (200 U / 20 µl) |
| Temperature and time | E | 60 min at 37°C; 2 min at 95°C |
| Manufacturer of reagents and catalogue numbers | D | Specified in “Complete reaction conditions”. |
| C_q_ with 🡪 without reverse transcription | D | The signal of the amplification plot without reverse transcriptase was very late and there was a high C_q_ value difference between the -RT control and all cDNA samples.  **GAPDH:** 21🡪none; **PPIB:** 24🡪none; **YWHAZ:** 24🡪38; **POLR2A:** 27🡪43; **TBP:** 29🡪40; **EEF1A1:** 19🡪37; **RPLP0:** 22🡪37; **RNA18S5:** 10🡪34; **RPL22**: 23🡪none |
| Storage conditions of cDNA | D | -20°C |
| **qPCR protocol** | | |
| Complete reaction conditions | E | RT-qPCR amplification was performed by using a Mastercycler^®^ ep realplex-S thermocycler (Eppendorf AG, Hamburg, Germany) in combination with 96 well PCR plates (Biozym Scientific) and BZO Seal Filmcover sheeting (Biozym Scientific). For each reaction 7.5 µl SYBR^®^Green JumpStart™ Taq ReadyMix™ (Sigma–Aldrich, consisting of Tris–HCl (20 mM, pH 8.3), KCl (100 mM), MgCl_2_ (7 mM), dNTPs (0.4 mM per dATP, dCTP, dGTP, dTTP), stabilizers, Taq-DNA-polymerase (0.05 U/µl), JumpStart™ Taq antibody and SYBR^®^Green I), as well as 1.5 µl of the respective cDNA solution (diluted 1:10) and 0.75 µl of the respective primer were pipetted. Nuclease-free H_2_O (Carl-Roth) was added to a total volume of 15 µl. We amplified the cDNA in triplets (technical replicates) per candidate reference gene and on the same qPCR plate per biological replicate in 45 cycles (initial heat activation 95°C/5 min, per cycle 95°C/10 s denaturation, 60°C/8 s annealing, 72°C/8 s extension,). At the end of each extension step SYBR^®^Green I fluorescence was measured at 521 nm. For each biological replicate all genes were amplified in triplet on the same qPCR plate to minimize biasing effects of possible inter-run variations on relative reference gene stability assessment. |
| Reaction volume and amount of cDNA/DNA | E | **Reaction volume:** 15 µl; **Amount of cDNA:** 1.5 µl of an 1:10 dilution of the cDNA stock solution |
| Primer, (probe), Mg_2_, and dNTP concentrations | E | 3.75 pmol/primer; 3.5 mM MgCl_2_; 0.2 mM dNTP; 50 mM KCl |
| Polymerase identity and concentration | E | Taq-DNA polymerase in a final concentration of 0.025 U/µl (SYBR^®^Green JumpStart™ Taq ReadyMix™, Sigma–Aldrich^®^, S4438, St. Louis, MI, USA) |
| Buffer/kit identity and manufacturer | E | SYBR^®^Green JumpStart™ Taq ReadyMix™ (Sigma–Aldrich^®^, S4438, St. Louis, MI, USA) |
| Exact chemical composition of the buffer | D | 20 mM Tris–HCl, pH 8.3, final concentration 10 mM |
| Additives (SYBR Green I, DMSO, and so forth) | E | SYBR Green I, stabilizers, JumpStart Taq antibody, KCl , MgCl_2_ |
| Manufacturer of plates/tubes and catalogue number | D | 96 well PCR plates (TW-MT, 712282, Biozym Scientific GmbH, Hessisch Oldendorf, Germany) in combination with BZO Seal Filmcover sheeting (712350, Biozym Scientific GmbH) |
| Complete thermocycling parameters | E | Initial heat activation 95°C/5min; per cycle 95°C/10s denaturation, 60°C/8s annealing, 72°C/8s extension |
| Reaction setup (manual/robotic) | D | manual |
| Manufacturer of qPCR instrument | D | Mastercycler^®^ ep realplex-S thermocycler (Eppendorf AG, Hamburg, Germany) |
| **qPCR validation** | | |
| Evidence of optimization | D | Primer optimization was evidenced by melting curve analysis and agarose gel electrophoresis (specifity), qPCR efficiency, technical reliability and in silico secondary structure analysis of primers and amplicons. Melting temperatures T_m_ of primers as validated by the manufacturer Eurofins MWG Operon LLC (Huntsville, AL, USA; High Purity Salt Free Purification HPSF^®^) are provided in Table 1. |
| Specifity (gel, sequence, melt or digest) | E | Specific amplification of tested reference genes was assessed by agarose gel electrophoreses (single band, correct size) and a specific peak in melting curve analysis (95°C for 15s, 60°C for 15s, then continuous temperature increase to 95°C and fluorescence measurement for 20 min; Supplementary Data 2). For each primer pair and qPCR run we also tested a no-template-control (NTC) without cDNA and a -RT control (cDNA synthesis without enzyme reverse transcriptase added) on the same plate to exclude possible bias by unspecific amplification (primer dimers, contaminating or genomic DNA). |
| For SYBR Green I, C_q_ of the NTC | E | The signal of the amplification plot during efficiency analysis for standard curve generation was very late and there was a high C_q_ value difference between the negative control and all cDNA dilutions.  **GAPDH:** 39; **PPIB:** none; **YWHAZ:** none; **POLR2A:** 37; **TBP:** none; **EEF1A1:** none; **RPLP0:** none; **RNA18S5:** 34; **RPL22:** none. |
| Calibration curves with slope and y intercept | E | **GAPDH:** y=1E+9e^-0.659x^, slope: -3.480; **PPIB:** y=5E+9e^-0.651x^, slope: -3.508; **YWHAZ:** y=6E+9e^-0.651x^, slope: -3.488; **POLR2A:** y=4E+10e^-0.651x^, slope: -3.520; **TBP:** y=3E+12e^-0.649x^, slope: -3.538; **EEF1A1:** y=7E+9e^-0.685x^, slope: -3.315; **RPLP0:** y=2E+9e^-0.646x^, slope: -3.509; **RNA18S5:** y=2E+6e^0.677x^, slope: -3.319; **RPL22:** y=1E+10e^-0.671x^, slope: -3.403 as already shown before (Kirschneck et al. 2017) |
| PCR efficiency calculated from slope | E | **GAPDH:** 93.8%; **PPIB:** 92.8%; **YWHAZ:** 93.5%; **POLR2A:** 92.3%; **TBP:** 91.7%; **EEF1A1:** 100.3%; **RPLP0:** 92.7%; **RNA18S5:** 100.1%; **RPL22:** 96.7% as already shown before (Kirschneck et al. 2017) |
| R^2^ of calibration curve | E | **GAPDH:** 0.9998; **PPIB:** 0.9996; **YWHAZ:** 0.9993; **POLR2A:** 0.9984; **TBP:** 0.9974; **EEF1A1:** 0.9951; **RPLP0:** 0.9992; **RNA18S5:** 0.9974; **RPL22:** 0.9949 as already shown before (Kirschneck et al. 2017) |
| Linear dynamic range (LDR) | E | The linear dynamic range (LDR) included the used 1:10 cDNA dilution in all cases and ranged from 3x log_10_ (cDNA stock dilution 1:10 – 1:10^3^) to 6x log_10_ (cDNA stock dilution 1:10 – 1:10^6^) for the individual genes (primer pairs) as already shown before (Kirschneck et al. 2017). Standard curves were calculated only considering dilutions within the LDR as already shown before (Kirschneck et al. 2017) |
| C_q_ variation at LOD | E | **GAPDH:** SD=0.952; **PPIB:** SD=1.77; **YWHAZ:** SD=1.696; **POLR2A:** SD=1.004; **TBP:** SD=0.561; **EEF1A1:** SD=0.405; **RPLP0:** SD=0.176; **RNA18S5:** SD=0.000; **RPL22:** SD=0.202 as already shown before (Kirschneck et al. 2017) |
| Evidence for LOD | E | Not detectable C_q_ value for ≥ 1 of the technical replicates (triplet) at the corresponding cDNA dilution level indicates LOD at the previous, more concentrated dilution level. LOD for all genes (primer pairs) detected at a cDNA quantity equivalent to ≤1 pg RNA, except for TBP with an LOD of 100 pg RNA equivalent (weak signal at 10 pg and 1 pg), as already shown before (Kirschneck et al. 2017) |
| If multiplex, efficiency and LOD of each assay | E | Not applicable. |
| **Data analysis** | | |
| qPCR analysis program (source, version) | E | Mastercycler ep realplex software, version 2.2 (Eppendorf AG, Hamburg, Germany) |
| Method of C_q_ determination | E | Second derivative maximum method (CalqPlex algorithm, Automatic Baseline, Drift Correction On) |
| Outlier identification and disposition | E | For analysis none of the C_q_ values was discarded. |
| Results for NTCs | E | The signal of the amplification plot was very late and there was a high C_q_ value difference between the negative control and all cDNA samples **GAPDH:** 39; **PPIB:** none; **YWHAZ:** none; **POLR2A:** 37; **TBP:** none; **EEF1A1:** none; **RPLP0:** none; **RNA18S:** 34; **RPL22:** none. |
| Justification of number and choice of reference genes | E | Aim of this study - identification of optimal number and choice of reference genes for normal and osteoarthritis synovial fibroblasts under physiological conditions and after compressive force application. |
| Description of normalization method | E | Samples were not normalized, since apart from the reference genes no target genes were quantified. For P4HA1 we normalized to untreated synovial fibroblasts from healthy donors and using all respective candidate housekeeping genes in comparison. |
| Number and concordance of biological replicates | D | Experiments were repeated twice (N = 2) with at least 3 biological replicates per experiment (n = 6) for each experimental group |
| Number and stage (RT or qPCR) of technical replicates | E | qPCR reactions were performed in triplets (technical replicates n = 3). |
| Repeatability (intraassay variation) | E | The maximum SD (of the mean) across all biological replicates (n=24) of the means of C_q_ from the three technical replicates was ≤ 0.55 in all instances.  **GAPDH:** 0.11; **PPIB:** 0.52; **YWHAZ:** 0.16; **POLR2A:** 0.09; **TBP:** 0.33; **EEF1A1:** 0.54; **RPLP0:** 0.17; **RNA18S5:** 0.47; **RPL22:** 0.32. |
| Reproducibility (interassay variation, CV) | D | High biological reproducibility was achieved as evidenced by the low SD of raw C_q_ values for all genes and experimental groups tested (see Figure 2, Supplementary Table 3). |
| Power analysis | D | The number of biological replicates (n = 6) was based on previous studies and corresponds to the number of replicates generally used in cell culture RT-qPCR experiments. |
| Statistical methods for results significance | E | All biological samples (n = 6) were measured in triplicate (n = 3) and an arithmetic mean of each C_q_ triplett used for further analysis. The stability of each tested reference gene was calculated with four different mathematical algorithms: geNorm, NormFinder, BestKeeper and the comparative ΔC_q_ method. Stability calculations were done with the official Microsoft-Excel-based software applets for geNorm, NormFinder and BestKeeper according to developers’ instructions. For the comparative ΔC_q_ method manual calculations were performed. The geNorm and NormFinder algorithms require the transformation of the raw C_q_ data to linear scale expression quantities Q corresponding to the qPCR efficiency (E) of each gene: Q = E^-(Cqmin-Cqsample)^ with the lowest C_q_ value corresponding to a quantity of 1 for each candidate reference gene. The genes were ranked according to their stability values (geNorm: M, NormFinder: ρ_ig_/σ_i_, deltaCT: mean SD of ∆C_q_; BestKeeper: Pearson’s r) for each algorithm and each experimental condition as well as combined experimental conditions and a rank sum of all algorithms calculated per gene for final stability assessment with the smallest rank sum indicating the most stable reference gene. Also a pooled overall ranking for all experimental conditions was calculated. The geNorm algorithm was used to calculate the ideal number of reference genes for reliable RT-qPCR normalization. If pairwise variation (V_n_/V_n+1_) between two sets of reference genes with one set including an additional reference gene was ≤0.15, this additional gene was deemed unnecessary for normalization. To assess ranking variations between the algorithms, we used IBM SPSS Statistics^®^ 23 (IBM, Armonk, NY, USA) to create a correlation matrix of bivariate correlations (Pearson´s correlation coefficient r, normality confirmed by Shapiro-Wilk tests and histogram evaluation) of the overall pooled stability values as calculated by two respective algorithms. |
| Software (source, version) | E | Microsoft Excel 2010 (Microsoft Corporation, Redmond, USA);  IBM SPSS Statistics^®^ 23 (IBM, Armonk, NY, USA) |
| C_q_ or raw data submission | D | Raw C_q_ values are provided in Supplementary Table 3. |
| **qPCR target information** | | |
| Gene symbol | E | Provided in Table 1. We based our primer design on the officially registered target gene nucleotide sequences from the NCBI Nucleotide database (GeneBank, access: http://www.ncbi.nlm.nih.gov/nuccore). |
| Sequence accession number | E |  |
| Location of amplicon | D | Provided in Table 1. |
| Amplicon length | E | Provided in Table 1. Target amplicon sequences were chosen to range from 60 to 150 bp with a GC content of 35–65%. |
| In silico specificity screen (BLAST, and so on) | E | Provided in Table 1. In-silico specify of constructed primers was ensured by PrimerBLAST (National Center for Biotechnology Information, Bethesda MD, USA, https://www.ncbi.nlm.nih.gov/tools/primer-blast; RefSeq mRNA, Splice variants allowed, Max. Product Size: 4000) and cross-checked using the UCSC in-silico-PCR Genome Browser (Dec. 2013 GRCh38/hg38; UCSC Genes; Max. Product Size: 4000; Min. Perfect Match: 15; Min. Good Match: 15; Jim Kent, http://genome-mirror.genomedk.au.dk/cgi-bin/hgPcr). Intron-flanking primer pairs were designed to prevent a co-amplification of genomic DNA and checked in silico for sufficient absence of hairpin structures and dimer formation at annealing temperature (∆G ≥ -3,5 kcal/mol, BeaconDesigner™ Free Edition, Premier BioSoft International, Palo Alto, CA, USA, http://www.premierbiosoft.com/qOligo/Oligo.jsp?PID=1). |
| Pseudogenes, retropseudogenes or other homologs | D | Sequence alignment, possible splicing and targeted transcript variants as well as absence of targeted pseudogenes, retropseudogenes or other homologs were assessed upon primer construction by NCBI PrimerBLAST (National Center for Biotechnology Information, Bethesda MD, USA, https://www.ncbi.nlm.nih.gov/tools/primer-blast) and PrimerCheck (SpliceCenter der Genomics and Bioinformatics Group, LMP, CCR, NCI, http://projects.insilico.us/SpliceCenter/PrimerCheck.jsp). |
| Sequence alignment | D |  |
| Secondary structure analysis of amplicon | D | No secondary structures present at annealing temperature (60°C) were detected as determined in silico by UNAFold (http://eu.idtdna.com/UNAFold?, Suboptimality 50%; Integrated DNA Technologies Inc., Coralville, IA, USA). |
| Location of each primer by exon or intron | E | Provided in Table 1. |
| What splice variants are targeted | E | Provided in Table 1. |
| **qPCR oligonucleotides** | | |
| Primer sequences | E | Provided in Table 1. |
| RTPrimerDB identification number | D | Not applicable, primers were constructed and validated by the authors. |
| Probe sequences | D | Not applicable. |
| Location and identity of any modifications | E | Primers received no terminal or other modifications. |
| Manufacturer of oligonucleotides | D | Primers were synthesized by Eurofins MWG Operon LLC (Huntsville, AL, USA). |
| Purification method | D | Primers were purified by High Purity Salt Free Purification HPSF^®^ (Eurofins MWG Operon LLC). |

Reference

Kirschneck, Christian; Batschkus, Sarah; Proff, Peter; Köstler, Josef; Spanier, Gerrit; Schröder, Agnes (2017): Valid gene expression normalization by RT-qPCR in studies on hPDL fibroblasts with focus on orthodontic tooth movement and periodontitis. In: *Scientific reports* 7 (1): 14751. DOI: 10.1038/s41598-017-15281-0.
