# Supplementary material for: Housekeeping gene validation for RT-qPCR studies on synovial fibroblasts derived from healthy and osteoarthritic patients with focus on mechanical loading
Source: PLoS One. 2019 Dec 6;14(12):e0225790. doi: 10.1371/journal.pone.0225790 (PMC6897414; doi:10.1371/journal.pone.0225790)
Supplement: S3 Table — (DOCX) [file pone.0225790.s003.docx]

**S3 Table. Raw C_q_ values of RT-qPCR for the two experimental groups and nine potentially suitable housekeeping genes.**

| **RAW C_q_ values** | | Gene | Gene | Gene | Gene | Gene | Gene | Gene | Gene | Gene |
| --- | --- | --- | --- | --- | --- | --- | --- | --- | --- | --- |
| **Experiment** | **sample** | **EEF1A1** | **GAPDH** | **POLR2A** | **PPIB** | **RNA18S** | **RPL22** | **RPLP0** | **TBP** | **YWHAZ** |
| hSF02 | 1 | 19.78 | 21.00 | 26.45 | 23.68 | 10.27 | 23.97 | 21.94 | 29.34 | 23.70 |
| hSF02 | 2 | 20.12 | 21.32 | 26.81 | 24.06 | 10.52 | 24.13 | 22.19 | 29.72 | 24.08 |
| hSF02 | 3 | 19.73 | 21.02 | 26.48 | 23.63 | 10.30 | 23.68 | 21.92 | 29.42 | 23.73 |
| hSF02 | 4 | 19.73 | 21.03 | 26.78 | 23.57 | 10.12 | 23.84 | 21.82 | 29.17 | 23.92 |
| hSF02 | 5 | 19.74 | 21.16 | 26.89 | 23.90 | 9.96 | 24.04 | 21.94 | 29.30 | 23.98 |
| hSF02 | 6 | 19.53 | 20.99 | 26.51 | 23.42 | 9.96 | 23.65 | 21.74 | 29.02 | 23.66 |
| hSF02 | 7 | 19.34 | 20.73 | 26.45 | 23.43 | 9.72 | 23.21 | 21.50 | 28.88 | 23.10 |
| hSF02 | 8 | 19.55 | 20.99 | 26.75 | 23.39 | 10.03 | 23.14 | 21.79 | 29.34 | 23.40 |
| hSF02 | 9 | 19.86 | 20.88 | 26.53 | 23.50 | 10.33 | 23.61 | 21.82 | 29.01 | 23.29 |
| hSF02 | 10 | 19.37 | 20.85 | 27.03 | 23.84 | 9.93 | 23.25 | 21.69 | 29.06 | 23.62 |
| hSF02 | 11 | 19.21 | 20.87 | 26.81 | 23.83 | 9.46 | 23.31 | 21.63 | 29.08 | 23.46 |
| hSF02 | 12 | 19.78 | 21.01 | 26.71 | 24.07 | 10.64 | 24.43 | 21.73 | 28.88 | 23.50 |
| hSF06 | 1 | 19.40 | 20.95 | 26.36 | 23.63 | 10.13 | 23.40 | 21.40 | 29.00 | 23.79 |
| hSF06 | 2 | 19.09 | 20.97 | 26.16 | 23.38 | 9.44 | 23.22 | 21.46 | 28.99 | 23.74 |
| hSF06 | 3 | 19.26 | 21.11 | 26.26 | 23.77 | 9.58 | 23.52 | 21.61 | 29.03 | 23.91 |
| hSF06 | 4 | 19.73 | 21.64 | 26.82 | 24.84 | 9.70 | 23.67 | 21.89 | 29.20 | 24.28 |
| hSF06 | 5 | 19.58 | 21.37 | 26.89 | 23.92 | 9.88 | 23.69 | 21.56 | 29.12 | 23.99 |
| hSF06 | 6 | 19.10 | 20.51 | 26.45 | 22.98 | 8.97 | 23.13 | 21.11 | 28.43 | 23.54 |
| hSF06 | 7 | 19.17 | 20.76 | 26.38 | 23.36 | 9.10 | 23.22 | 21.53 | 28.85 | 23.17 |
| hSF06 | 8 | 19.63 | 21.01 | 26.56 | 23.49 | 9.67 | 23.68 | 21.79 | 28.92 | 23.32 |
| hSF06 | 9 | 19.43 | 20.53 | 26.18 | 23.19 | 9.47 | 23.29 | 21.40 | 28.50 | 22.96 |
| hSF06 | 10 | 19.09 | 20.80 | 26.45 | 23.52 | 9.03 | 23.30 | 21.29 | 28.69 | 23.11 |
| hSF06 | 11 | 19.09 | 20.94 | 26.70 | 23.75 | 9.11 | 23.24 | 21.28 | 28.82 | 23.34 |
| hSF06 | 12 | 19.15 | 20.90 | 26.27 | 23.46 | 9.15 | 23.08 | 21.12 | 28.65 | 23.06 |
| **C_q_ SD** |  | **0.08** | **0.07** | **0.31** | **0.16** | **0.13** | **0.13** | **0.10** | **0.05** | **0.33** |

C_q_ = quantification cycle; SD = standard deviation of group mean. Gene symbols see Table 1. *: Samples were excluded from ranking due to insufficient RIN value.
