# Supplementary material for: Housekeeping gene validation for RT-qPCR studies on synovial fibroblasts derived from healthy and osteoarthritic patients with focus on mechanical loading
Source: PLoS One. 2019 Dec 6;14(12):e0225790. doi: 10.1371/journal.pone.0225790 (PMC6897414; doi:10.1371/journal.pone.0225790)
Supplement: S4 Table — Statistics: unpaired t-test using GraphPad Prism version 8.0. (DOCX) [file pone.0225790.s004.docx]

**S4 Table.** Statistical differences given as p values in each experimental group (intragroup comparisons) between results of target gene analysis for *P4HA1, COL1A2, COX-2* and *IL-6* normalized to a single reference gene and results normalized to the geometric mean of *EEF1A1/RPLP0*. *Statistics:* unpaired t-test using GraphPad Prism version 8.0

**Target gene: *Prolyl-4-hydroxylase-alpha-1***

Prolyl-4-hydroxylase-alpha-1 P4HA1 gene expression normalized to EEF1A1/RPLP0 (geometric mean) statistically compared via unpaired t test to P4HA1 gene expression normalized to the respective genes in the table below for N-SF and OA-SF control and pressure groups

| *p value* | **N-SF** | | **OA-SF** | |
| --- | --- | --- | --- | --- |
|  | **control** | **pressure** | **control** | **pressure** |
| *EEF1A1* | 1.0000 | 0.5982 | 0.9240 | 0.9838 |
| *GAPDH* | 1.0000 | 0.1047 | 0.5542 | 0.7577 |
| *POLR2A* | 1.0000 | **0.0060**** | 0.4806 | 0.1718 |
| *PPIB* | 1.0000 | 0.2459 | 0.4118 | 0.3441 |
| *RNA18S* | 1.0000 | 0.2999 | 0.5782 | 0.9329 |
| *RPL22* | 1.0000 | 0.4996 | 0.4179 | 0.7469 |
| *RPLP0* | 1.0000 | 0.9344 | 0.8954 | 0.9853 |
| *TBP* | 1.0000 | 0.3555 | 0.3192 | 0.7583 |
| *YWHAZ* | 1.0000 | 0.0619 | 0.0537 | 0.6946 |

**Target gene: *Collagen-1-alpha-2***

Collagen-1-alpha-2 gene expression normalized to EEF1A1/RPLP0 (geometric mean) statistically compared via unpaired t test to Collagen-1-alpha-2 gene expression normalized to the respective genes in the table below for N-SF and OA-SF control and pressure groups

| *p value* | **N-SF** | | **OA-SF** | |
| --- | --- | --- | --- | --- |
|  | **control** | **pressure** | **control** | **pressure** |
| *EEF1A1* | 1.0000 | 0.7352 | 0.9036 | 0.9425 |
| *GAPDH* | 1.0000 | 0.5998 | 0.4300 | 0.6827 |
| *POLR2A* | 1.0000 | **0.0292*** | 0.5957 | **0.0245*** |
| *PPIB* | 1.0000 | 0.5347 | 0.3414 | 0.1836 |
| *RNA18S* | 1.0000 | 0.0731 | 0.3122 | 0.7982 |
| *RPL22* | 1.0000 | 0.6879 | 0.5203 | 0.6701 |
| *RPLP0* | 1.0000 | 0.7036 | 0.8809 | 0.9273 |
| *TBP* | 1.0000 | 0.1875 | 0.2104 | 0.5353 |
| *YWHAZ* | 1.0000 | **<0.0001***** | **0.0369*** | 0.2753 |

**Target gene: *Cyclooxygenase-2***

Cyclooxygenase-2 gene expression normalized to EEF1A1/RPLP0 (geometric mean) statistically compared via unpaired t test to Cyclooxygenase-2 gene expression normalized to the respective genes in the table below for N-SF and OA-SF control and pressure groups

| *p value* | **N-SF** | | **OA-SF** | |
| --- | --- | --- | --- | --- |
|  | **control** | **pressure** | **control** | **pressure** |
| *EEF1A1* | 1.0000 | 0.5704 | 0.5647 | 0.9533 |
| *GAPDH* | 1.0000 | 0.0915 | 0.7431 | 0.3802 |
| *POLR2A* | 1.0000 | 0.5247 | 0.5200 | 0.5820 |
| *PPIB* | 1.0000 | 0.0917 | 0.7989 | 0.9034 |
| *RNA18S* | 1.0000 | 0.6345 | 0.5838 | 0.8372 |
| *RPL22* | 1.0000 | 0.9459 | 0.9153 | 0.9615 |
| *RPLP0* | 1.0000 | 0.3182 | 0.5166 | 0.8898 |
| *TBP* | 1.0000 | 0.5494 | 0.1487 | 0.7016 |
| *YWHAZ* | 1.0000 | 0.5576 | **0.0386*** | 0.4698 |

**Target gene: *Interleukin-6***

Interleukin-6 gene expression normalized to EEF1A1/RPLP0 (geometric mean) statistically compared via unpaired t test to Interleukin-6 gene expression normalized to the respective genes in the table below for N-SF and OA-SF control and pressure groups

| *p value* | **N-SF** | | **OA-SF** | |
| --- | --- | --- | --- | --- |
|  | **control** | **pressure** | **control** | **pressure** |
| *EEF1A1* | 1.0000 | 0.7712 | 0.9568 | 0.9307 |
| *GAPDH* | 1.0000 | 0.5863 | 0.7801 | 0.2657 |
| *POLR2A* | 1.0000 | 0.0547 | 0.5726 | **0.0020**** |
| *PPIB* | 1.0000 | 0.5487 | 0.5839 | **0.0057**** |
| *RNA18S* | 1.0000 | 0.1531 | 0.5853 | 0.2803 |
| *RPL22* | 1.0000 | 0.8337 | 0.3032 | 0.6459 |
| *RPLP0* | 1.0000 | 0.7918 | 0.8953 | 0.8836 |
| *TBP* | 1.0000 | 0.3228 | 0.5647 | 0.4749 |
| *YWHAZ* | 1.0000 | 0.4947 | 0.2136 | 0.2386 |
