# Supplementary material for: Housekeeping gene validation for RT-qPCR studies on synovial fibroblasts derived from healthy and osteoarthritic patients with focus on mechanical loading
Source: PLoS One. 2019 Dec 6;14(12):e0225790. doi: 10.1371/journal.pone.0225790 (PMC6897414; doi:10.1371/journal.pone.0225790)
Supplement: S1 File — (PDF) [file pone.0225790.s005.pdf]

S1 File. RNA integrity analysis.

Experiment: hSF02

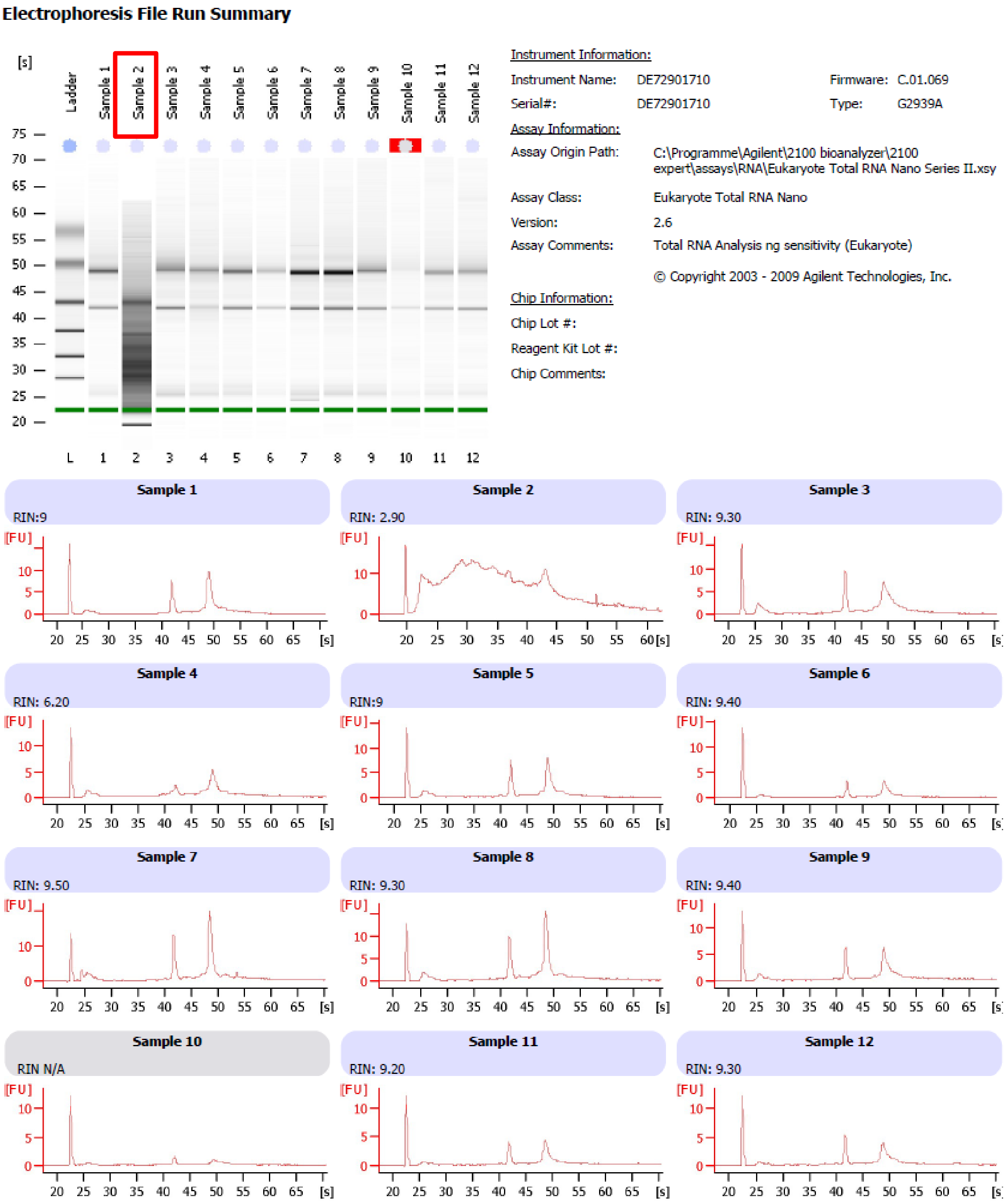

Electrophoresis File Run Summary (Chip Summary)

Sample Name	Sample Comment	Status	Result Label	Result Color
Sample 1		✔	RIN:9	
Sample 2		✔	RIN: 2.90	
Sample 3		✔	RIN: 9.30	
Sample 4		✔	RIN: 6.20	
Sample 5		✔	RIN:9	
Sample 6		✔	RIN: 9.40	
Sample 7		✔	RIN: 9.50	
Sample 8		✔	RIN: 9.30	
Sample 9		✔	RIN: 9.40	
Sample 10		✔	RIN N/A	
Sample 11		✔	RIN: 9.20	
Sample 12		✔	RIN: 9.30	
Ladder		✔	All Other Samples	

Sample 1

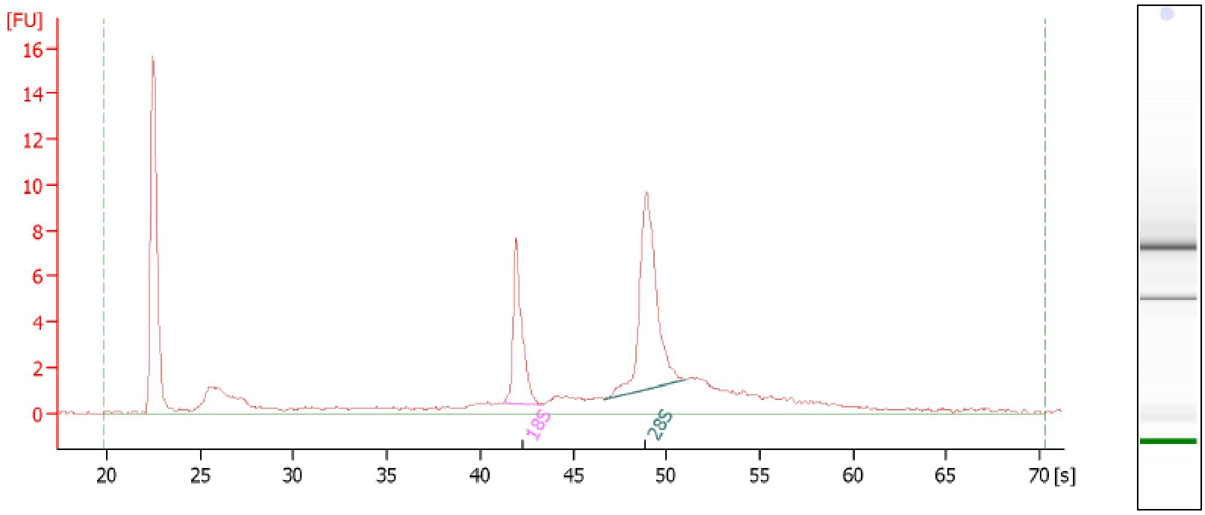

Overall Results for sample 1 : **Sample 1**

RNA Area:	78,4	RNA Integrity Number (RIN):	9 (B.02.08)
RNA Concentration:	31 ng/µl	Result Flagging Color:	
rRNA Ratio [28s / 18s]:	2,0	Result Flagging Label:	RIN:9

Fragment table for sample 1 : **Sample 1**

Name	Start Time [s]	End Time [s]	Area	% of total Area
18S	41,23	43,42	9,3	11,9
28S	46,63	51,01	18,8	23,9

Sample 2

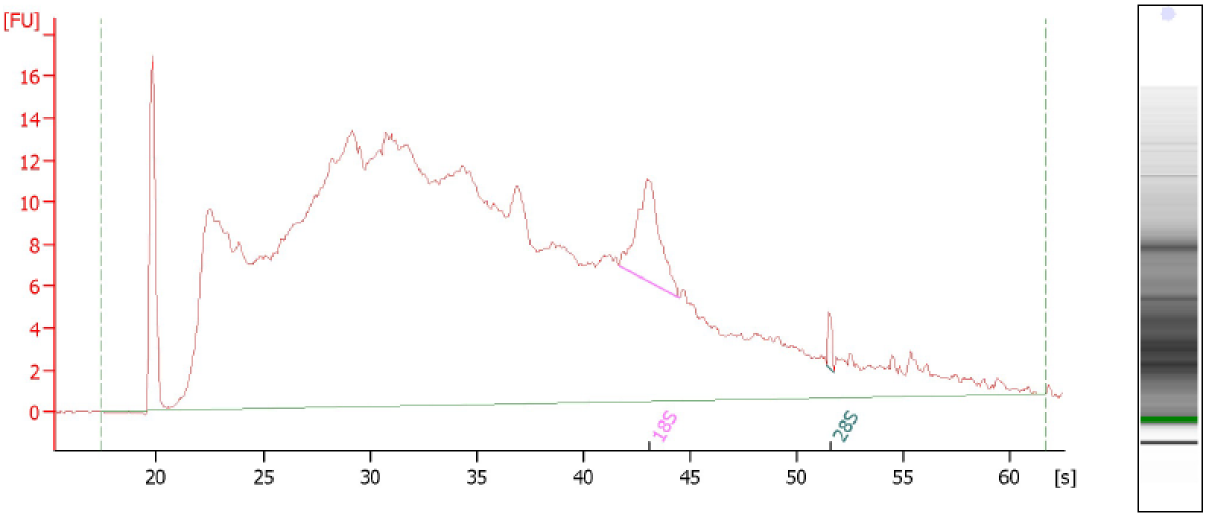

Overall Results for sample 2 : **Sample 2**

RNA Area:	685,0	RNA Integrity Number (RIN):	2.9 (B.02.08)
RNA Concentration:	275 ng/µl	Result Flagging Color:	
rRNA Ratio [28s / 18s]:	0,1	Result Flagging Label:	RIN: 2.90

Fragment table for sample 2 : **Sample 2**

Name	Start Time [s]	End Time [s]	Area	% of total Area
18S	41,65	44,46	13,9	2,0
28S	51,38	51,74	1,0	0,1

Sample 3

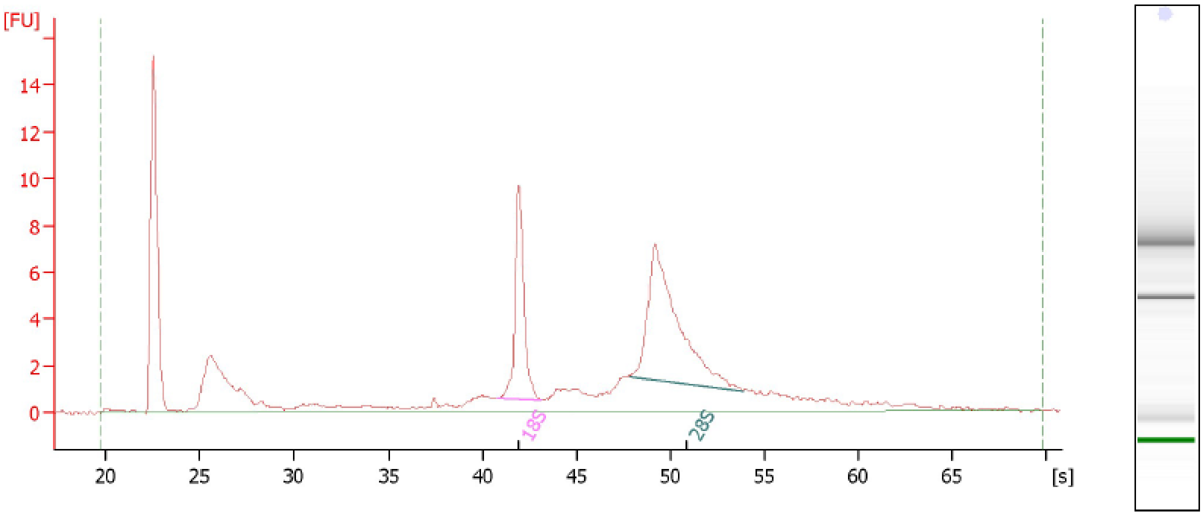

Overall Results for sample 3 : Sample 3

RNA Area:	96,3	RNA Integrity Number (RIN):	9.3 (B.02.08)
RNA Concentration:	39 ng/µl	Result Flagging Color:	
rRNA Ratio [28s / 18s]:	1,8	Result Flagging Label:	RIN: 9.30

Fragment table for sample 3 : Sample 3

Name	Start Time [s]	End Time [s]	Area	% of total Area
18S	40,80	43,18	11,6	12,0
28S	47,83	53,85	21,3	22,1

Sample 4

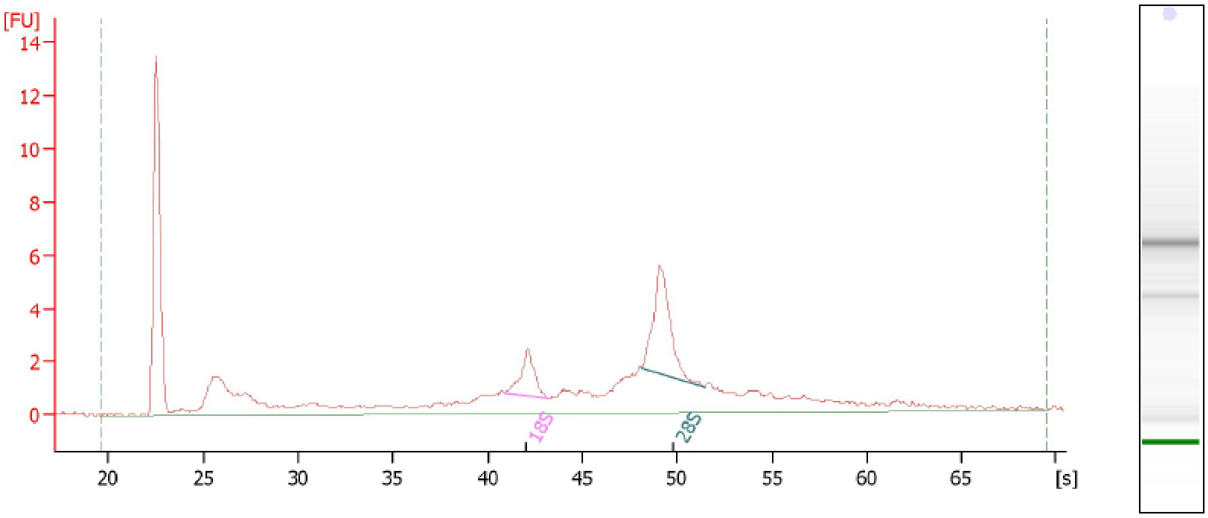

Overall Results for sample 4 : Sample 4

RNA Area:	71,6	RNA Integrity Number (RIN):	6.2 (B.02.08)
RNA Concentration:	29 ng/µl	Result Flagging Color:	
rRNA Ratio [28s / 18s]:	2,4	Result Flagging Label:	RIN: 6.20

Fragment table for sample 4 : Sample 4

Name	Start Time [s]	End Time [s]	Area	% of total Area
18S	40,92	43,24	3,4	4,7
28S	48,12	51,44	8,3	11,6

Sample 5

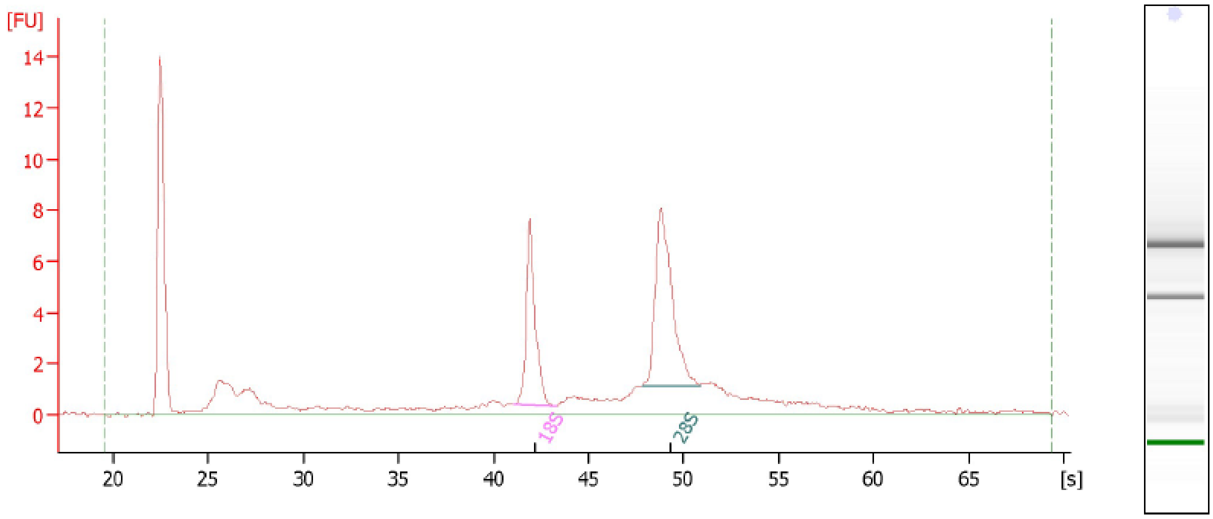

Overall Results for sample 5 : Sample 5

RNA Area:	71,9	RNA Integrity Number (RIN):	9 (B.02.08)
RNA Concentration:	29 ng/µl	Result Flagging Color:	
rRNA Ratio [28s / 18s]:	1,5	Result Flagging Label:	RIN:9

Fragment table for sample 5 : Sample 5

Name	Start Time [s]	End Time [s]	Area	% of total Area
18S	41,08	43,29	9,7	13,4
28S	47,81	50,88	14,6	20,3

Sample 6

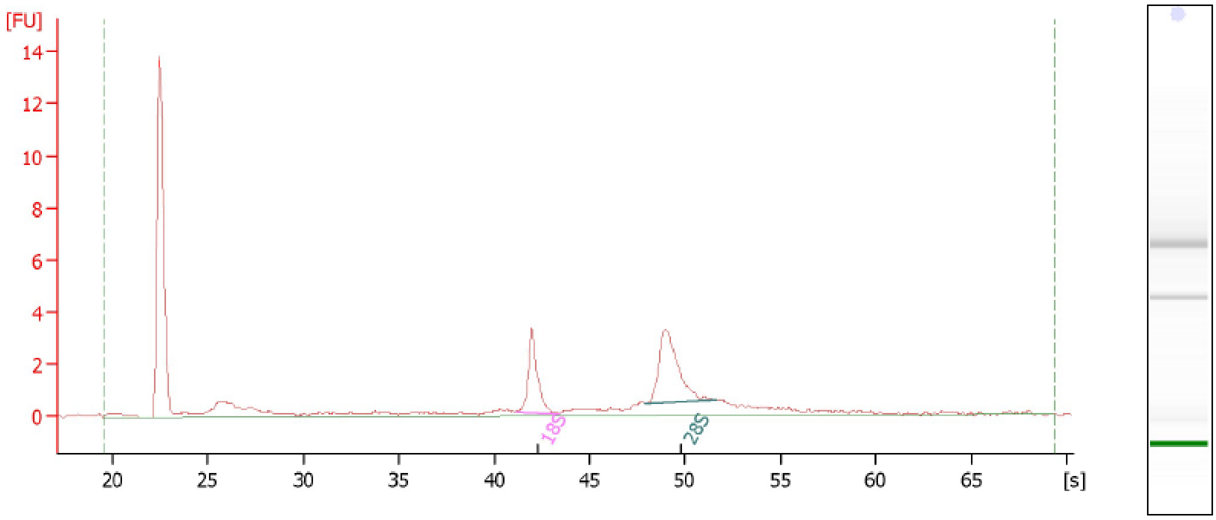

Overall Results for sample 6 : Sample 6

RNA Area:	34,0	RNA Integrity Number (RIN):	9.4 (B.02.08)
RNA Concentration:	14 ng/µl	Result Flagging Color:	
rRNA Ratio [28s / 18s]:	1,6	Result Flagging Label:	RIN: 9.40

Fragment table for sample 6 : Sample 6

Name	Start Time [s]	End Time [s]	Area	% of total Area
18S	41,13	43,39	4,3	12,7
28S	47,91	51,63	6,8	19,9

Sample 7

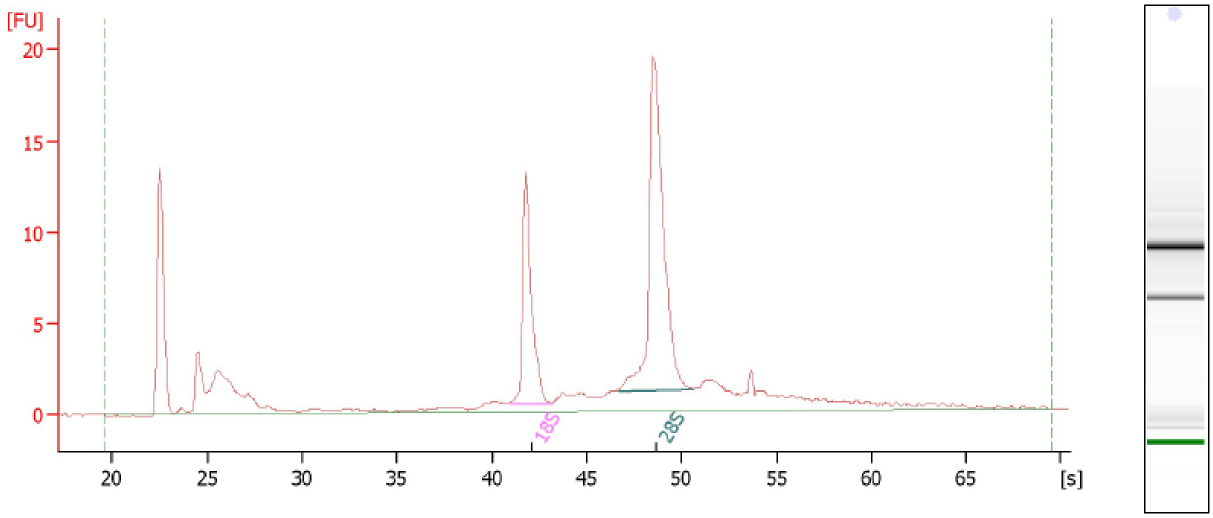

Overall Results for sample 7 : **Sample 7**

RNA Area:	115,9	RNA Integrity Number (RIN):	9.5 (B.02.08)
RNA Concentration:	47 ng/µl	Result Flagging Color:	
rRNA Ratio [28s / 18s]:	2,1	Result Flagging Label:	RIN: 9.50

Fragment table for sample 7 : **Sample 7**

Name	Start Time [s]	End Time [s]	Area	% of total Area
18S	40,97	43,19	15,6	13,5
28S	46,66	50,64	33,1	28,5

Sample 8

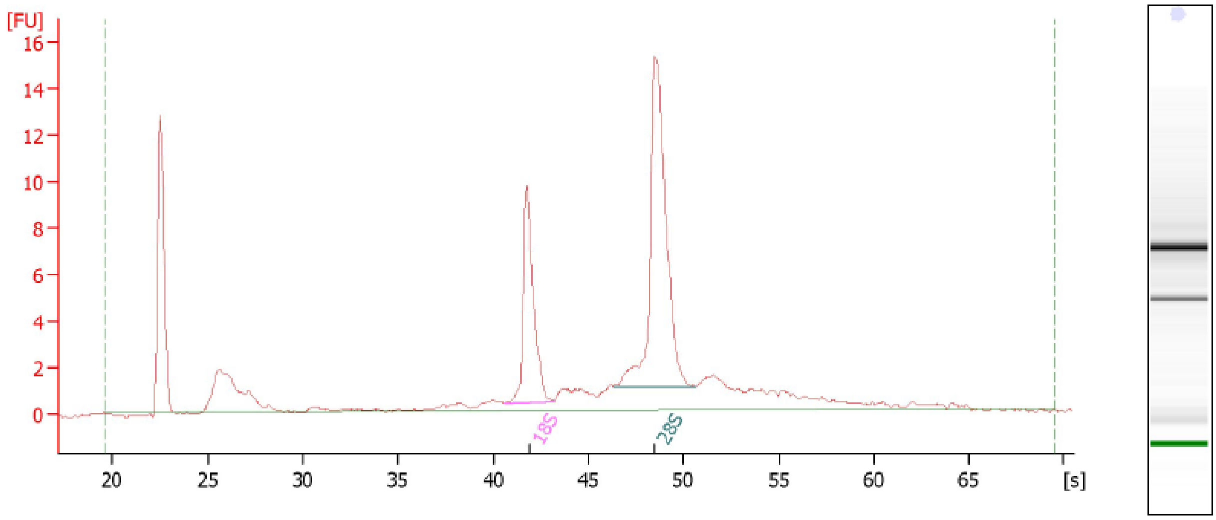

Overall Results for sample 8 : **Sample 8**

RNA Area:	90,6	RNA Integrity Number (RIN):	9.3 (B.02.08)
RNA Concentration:	36 ng/µl	Result Flagging Color:	
rRNA Ratio [28s / 18s]:	2,2	Result Flagging Label:	RIN: 9.30

Fragment table for sample 8 : **Sample 8**

Name	Start Time [s]	End Time [s]	Area	% of total Area
18S	40,62	43,19	13,1	14,4
28S	46,31	50,64	28,4	31,3

Sample 9

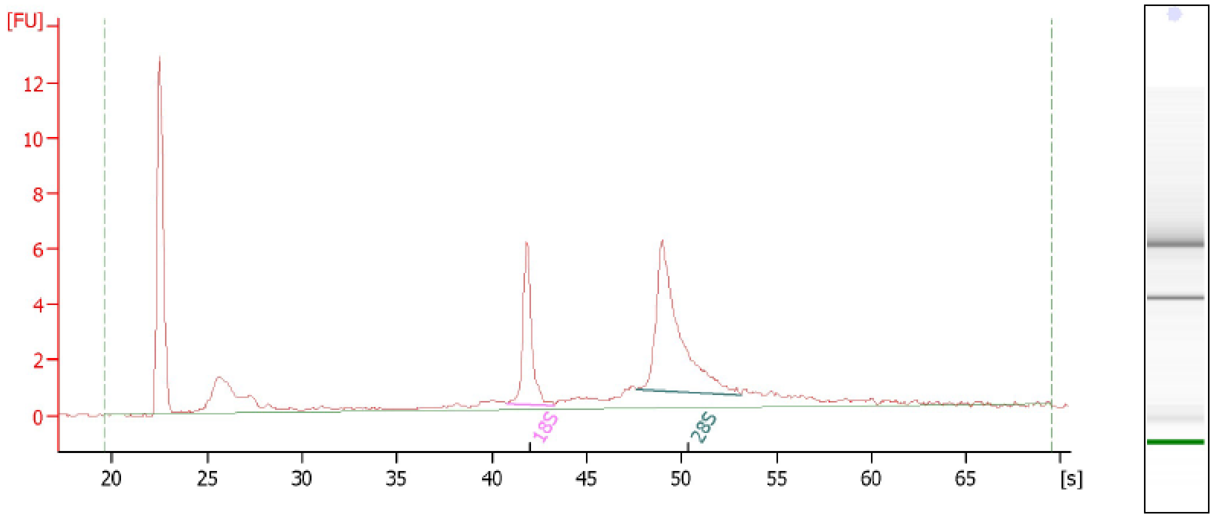

Overall Results for sample 9 : **Sample 9**

RNA Area:	53,9	RNA Integrity Number (RIN):	9.4 (B.02.08)
RNA Concentration:	22 ng/µl	Result Flagging Color:	
rRNA Ratio [28s / 18s]:	2,1	Result Flagging Label:	RIN: 9.40

Fragment table for sample 9 : **Sample 9**

Name	Start Time [s]	End Time [s]	Area	% of total Area
18S	40,82	43,29	7,1	13,2
28S	47,67	53,15	15,2	28,2

Sample 10

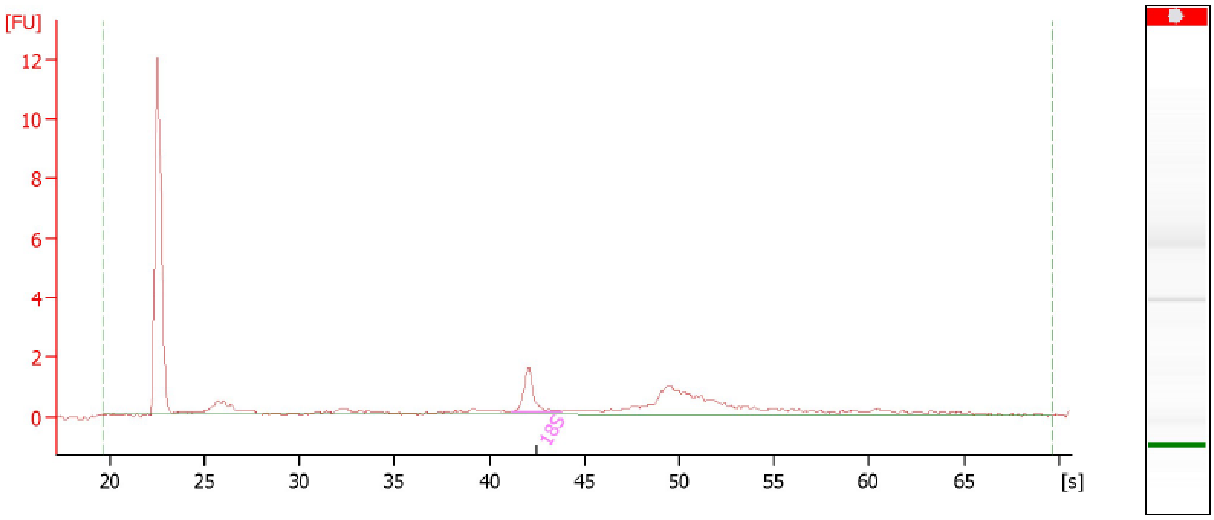

Overall Results for sample 10 : **Sample 10**

RNA Area:	19,7	RNA Integrity Number (RIN):	N/A (B.02.08)
RNA Concentration:	8 ng/µl	Result Flagging Color:	
rRNA Ratio [28s / 18s]:	0,0	Result Flagging Label:	RIN N/A

Fragment table for sample 10 : **Sample 10**

Name	Start Time [s]	End Time [s]	Area	% of total Area
18S	41,17	43,84	2,1	10,6

Sample 11

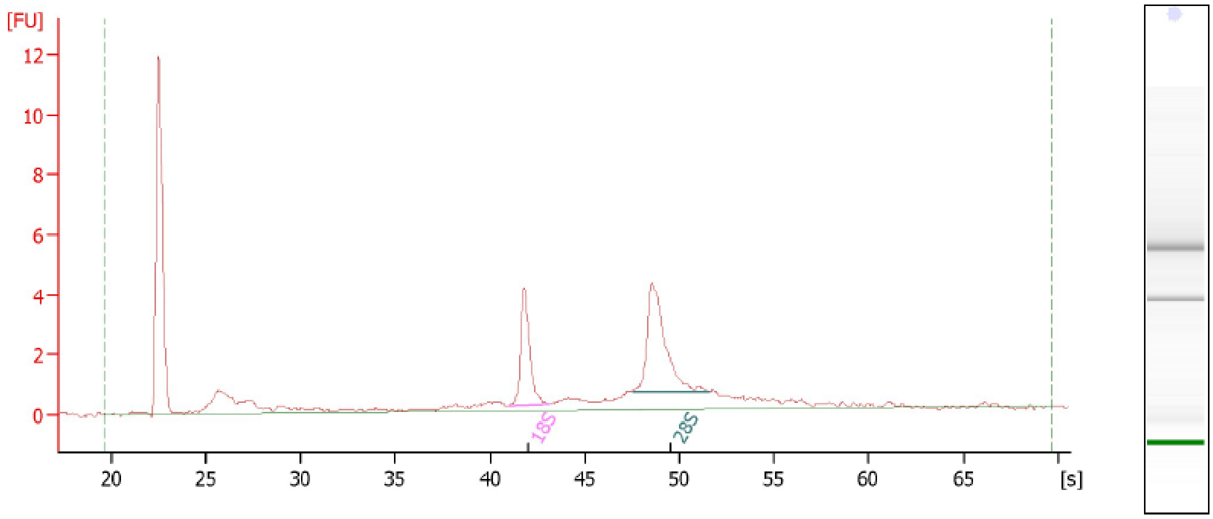

Overall Results for sample 11 : **Sample 11**

RNA Area:	38,2	RNA Integrity Number (RIN):	9.2 (B.02.08)
RNA Concentration:	15 ng/µl	Result Flagging Color:	
rRNA Ratio [28s / 18s]:	1,6	Result Flagging Label:	RIN: 9.20

Fragment table for sample 11 : **Sample 11**

Name	Start Time [s]	End Time [s]	Area	% of total Area
18S	41,01	43,13	5,0	13,1
28S	47,52	51,56	8,2	21,6

Sample 12

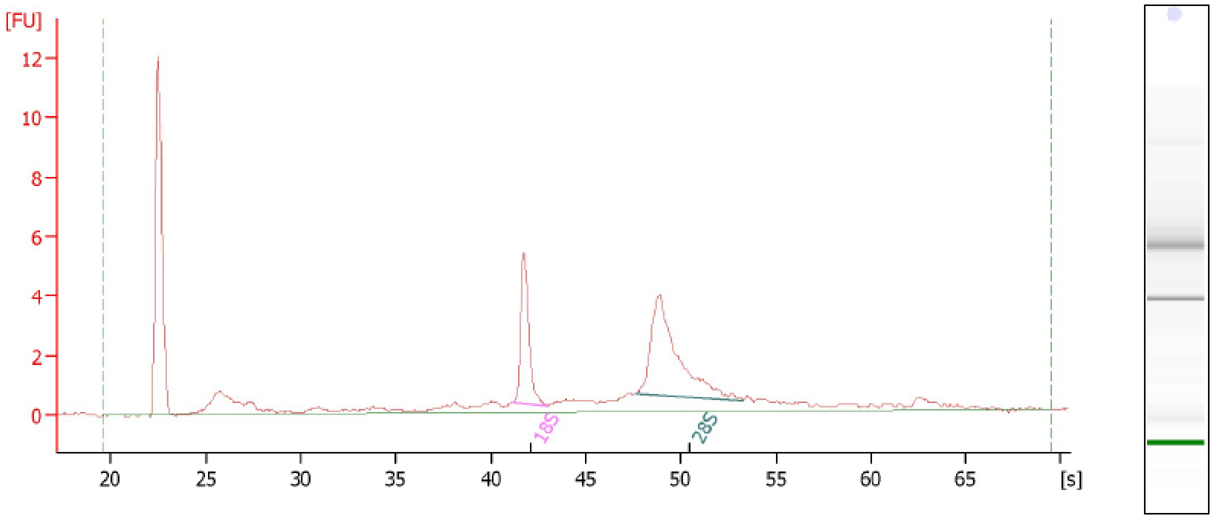

Overall Results for sample 12 : **Sample 12**

RNA Area:	46,1	RNA Integrity Number (RIN):	9.3 (B.02.08)
RNA Concentration:	19 ng/µl	Result Flagging Color:	
rRNA Ratio [28s / 18s]:	1,8	Result Flagging Label:	RIN: 9.30

Fragment table for sample 12 : **Sample 12**

Name	Start Time [s]	End Time [s]	Area	% of total Area
18S	41,12	43,09	5,9	12,9
28S	47,62	53,31	10,9	23,6

Experiment: hSF06

Electrophoresis File Run Summary

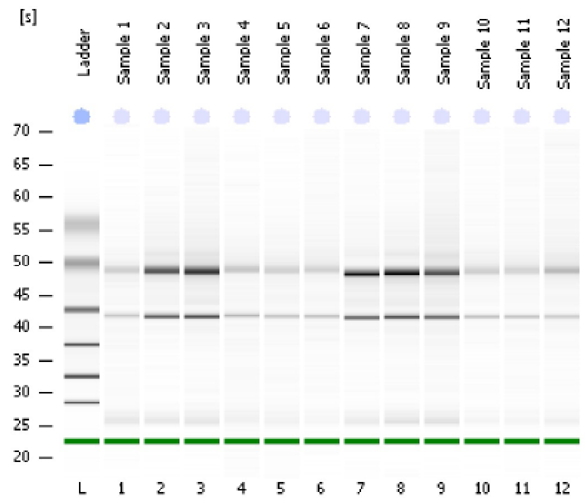

Instrument Information:

Instrument Name: DE72901710 Firmware: C.01.069

Serial#: DE72901710 Type: G2939A

Assay Information:

Assay Origin Path: C:\Programme\Agilent\2100 bioanalyzer\2100 expert\assays\RNA\Eukaryote Total RNA Nano Series II.xsy

Assay Class: Eukaryote Total RNA Nano

Version: 2.6

Assay Comments: Total RNA Analysis ng sensitivity (Eukaryote)

© Copyright 2003 - 2009 Agilent Technologies, Inc.

Chip Information:

Chip Lot #:

Reagent Kit Lot #:

Chip Comments:

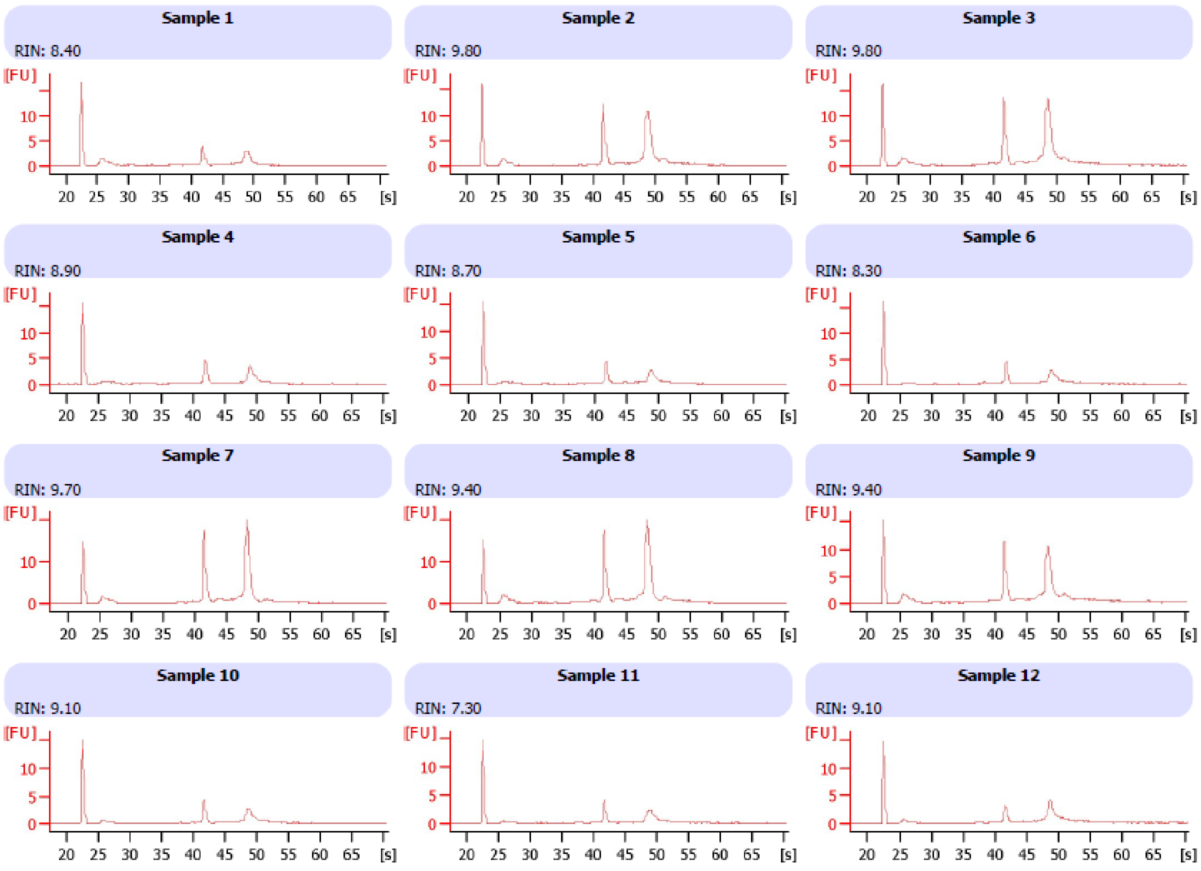

Electrophoresis File Run Summary (Chip Summary)

Sample Name	Sample Comment	Status	Result Label	Result Color
Sample 1		✔	RIN: 8.40	
Sample 2		✔	RIN: 9.80	
Sample 3		✔	RIN: 9.80	
Sample 4		✔	RIN: 8.90	
Sample 5		✔	RIN: 8.70	
Sample 6		✔	RIN: 8.30	
Sample 7		✔	RIN: 9.70	
Sample 8		✔	RIN: 9.40	
Sample 9		✔	RIN: 9.40	
Sample 10		✔	RIN: 9.10	
Sample 11		✔	RIN: 7.30	
Sample 12		✔	RIN: 9.10	
Ladder		✔	All Other Samples	

Sample 1

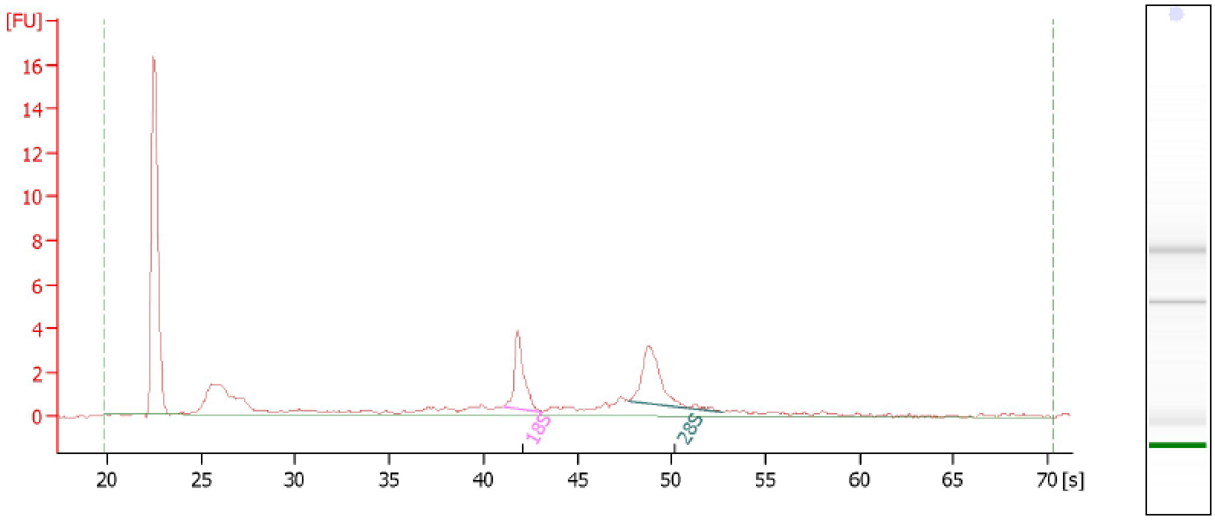

Overall Results for sample 1 : **<u>Sample 1</u>**

RNA Area:	41,6	RNA Integrity Number (RIN):	8.4 (B.02.08)
RNA Concentration:	19 ng/µl	Result Flagging Color:	
rRNA Ratio [28s / 18s]:	1,3	Result Flagging Label:	RIN: 8.40

Fragment table for sample 1 : **<u>Sample 1</u>**

Name	Start Time [s]	End Time [s]	Area	% of total Area
18S	41,08	43,17	4,9	11,8
28S	47,70	52,74	6,4	15,3

Sample 2

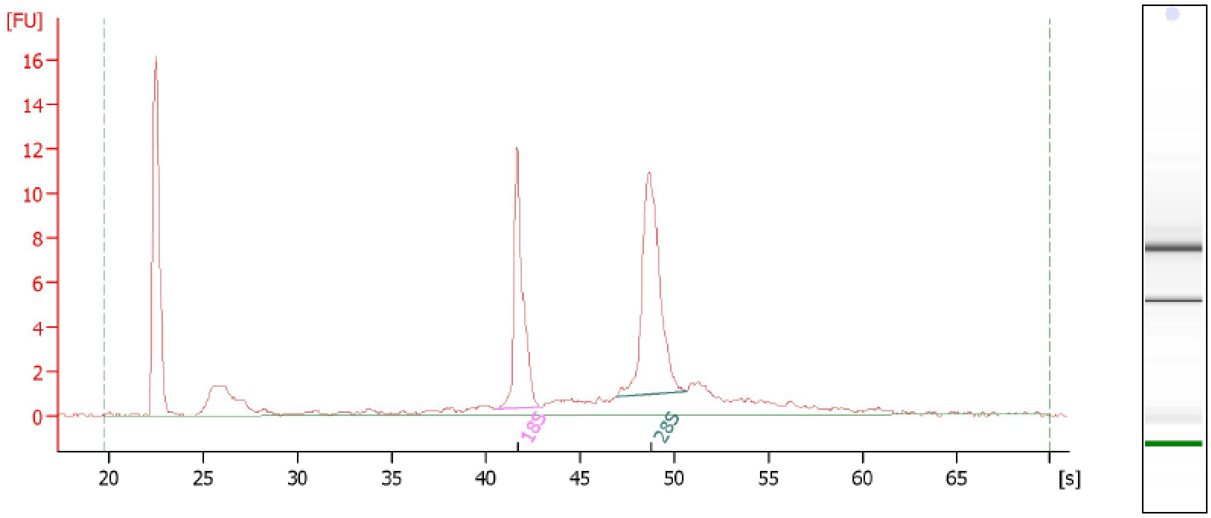

Overall Results for sample 2 : **<u>Sample 2</u>**

RNA Area:	78,3	RNA Integrity Number (RIN):	9.8 (B.02.08)
RNA Concentration:	37 ng/µl	Result Flagging Color:	
rRNA Ratio [28s / 18s]:	1,5	Result Flagging Label:	RIN: 9.80

Fragment table for sample 2 : **<u>Sample 2</u>**

Name	Start Time [s]	End Time [s]	Area	% of total Area
18S	40,59	42,87	15,1	19,3
28S	46,93	50,73	22,0	28,1

Sample 3

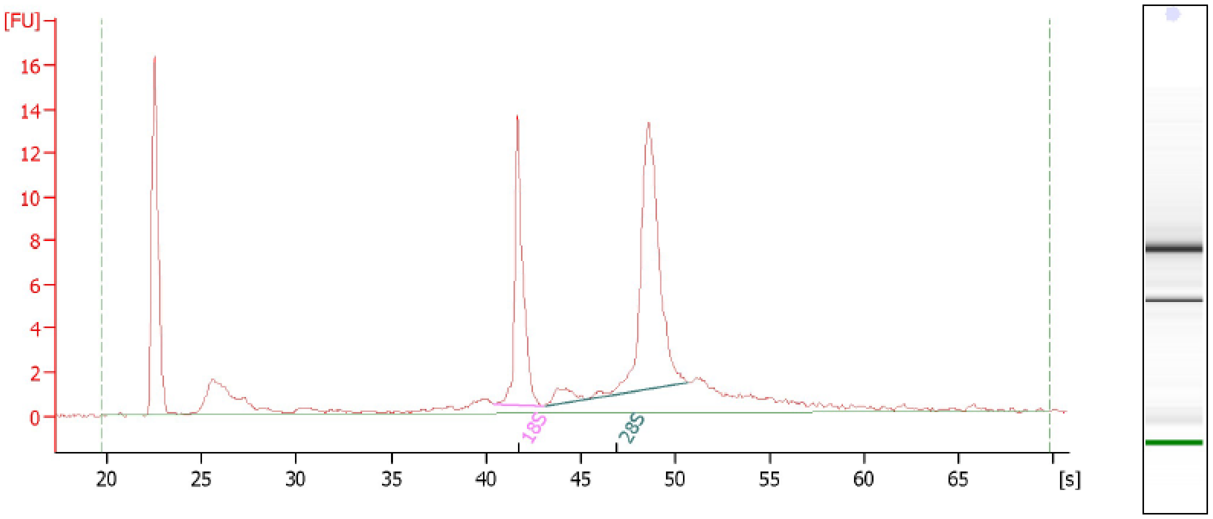

Overall Results for sample 3 : **Sample 3**

RNA Area:	94,2	RNA Integrity Number (RIN):	9.8 (B.02.08)
RNA Concentration:	44 ng/µl	Result Flagging Color:	
rRNA Ratio [28s / 18s]:	1,8	Result Flagging Label:	RIN: 9.80

Fragment table for sample 3 : **Sample 3**

Name	Start Time [s]	End Time [s]	Area	% of total Area
18S	40,45	43,03	16,1	17,1
28S	43,13	50,71	29,8	31,6

Sample 4

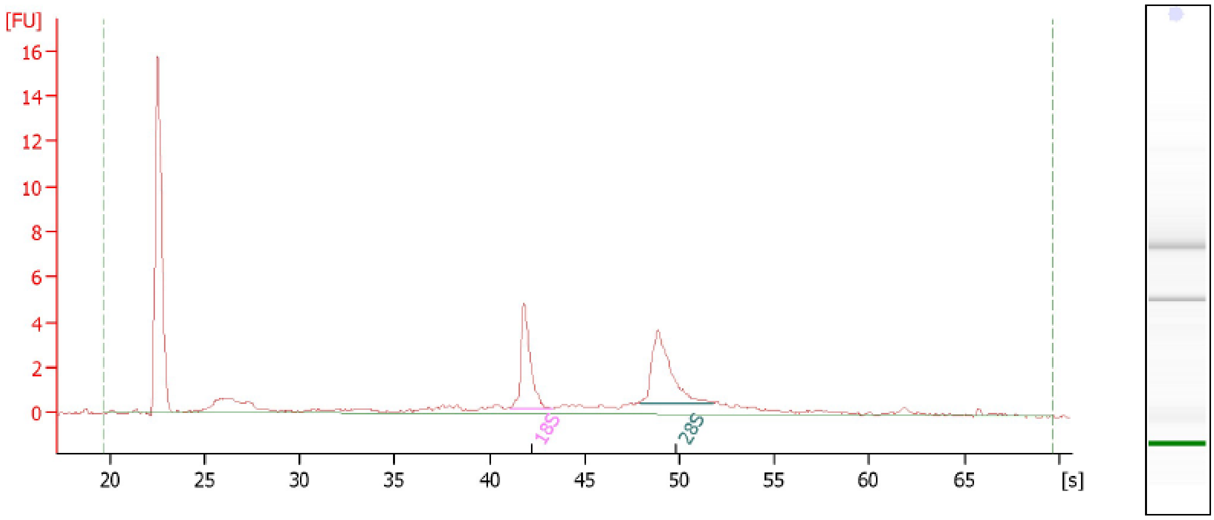

Overall Results for sample 4 : **Sample 4**

RNA Area:	41,4	RNA Integrity Number (RIN):	8.9 (B.02.08)
RNA Concentration:	19 ng/µl	Result Flagging Color:	
rRNA Ratio [28s / 18s]:	1,2	Result Flagging Label:	RIN: 8.90

Fragment table for sample 4 : **Sample 4**

Name	Start Time [s]	End Time [s]	Area	% of total Area
18S	41,07	43,34	6,0	14,5
28S	47,93	51,81	7,4	17,9

Sample 5

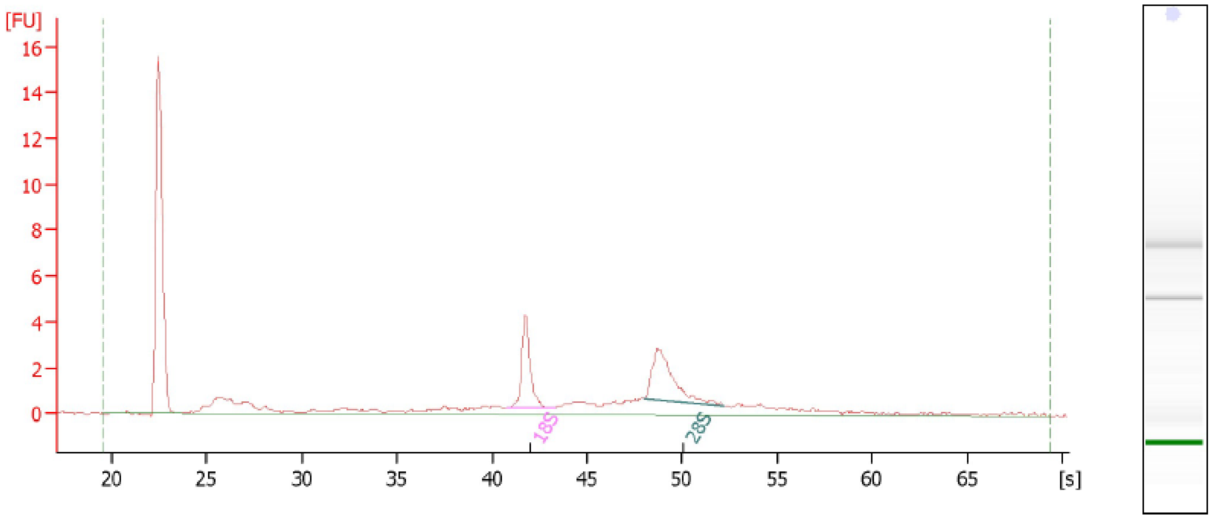

Overall Results for sample 5 : **Sample 5**

RNA Area:	41,4	RNA Integrity Number (RIN):	8.7 (B.02.08)
RNA Concentration:	19 ng/µl	Result Flagging Color:	
rRNA Ratio [28s / 18s]:	1,1	Result Flagging Label:	RIN: 8.70

Fragment table for sample 5 : **Sample 5**

Name	Start Time [s]	End Time [s]	Area	% of total Area
18S	40,78	43,29	5,1	12,2
28S	47,96	52,18	5,8	14,1

Sample 6

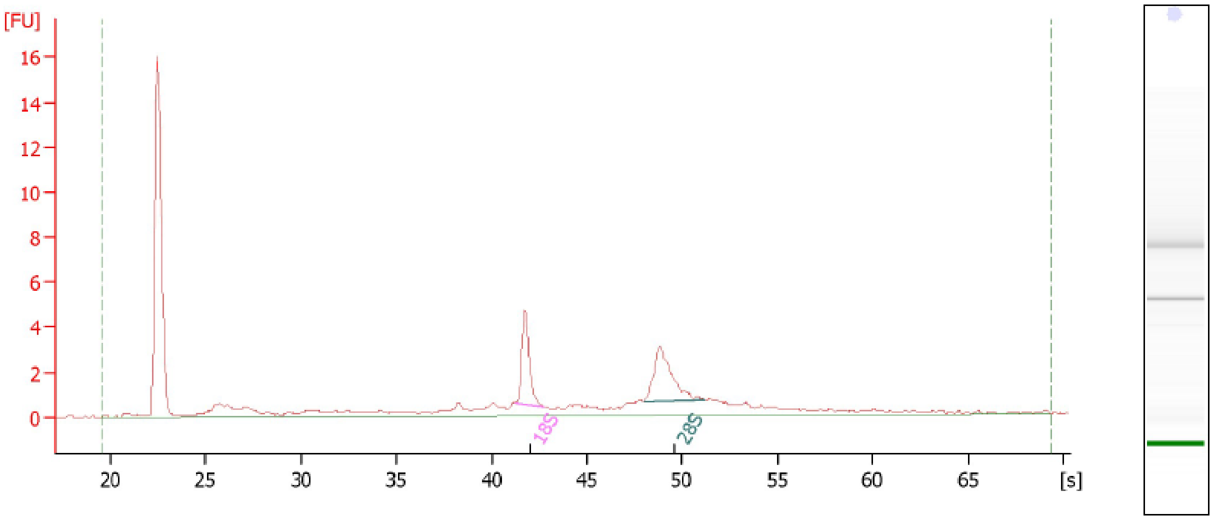

Overall Results for sample 6 : **Sample 6**

RNA Area:	41,2	RNA Integrity Number (RIN):	8.3 (B.02.08)
RNA Concentration:	19 ng/µl	Result Flagging Color:	
rRNA Ratio [28s / 18s]:	1,3	Result Flagging Label:	RIN: 8.30

Fragment table for sample 6 : **Sample 6**

Name	Start Time [s]	End Time [s]	Area	% of total Area
18S	41,23	42,74	4,3	10,4
28S	47,96	51,18	5,4	13,2

Sample 7

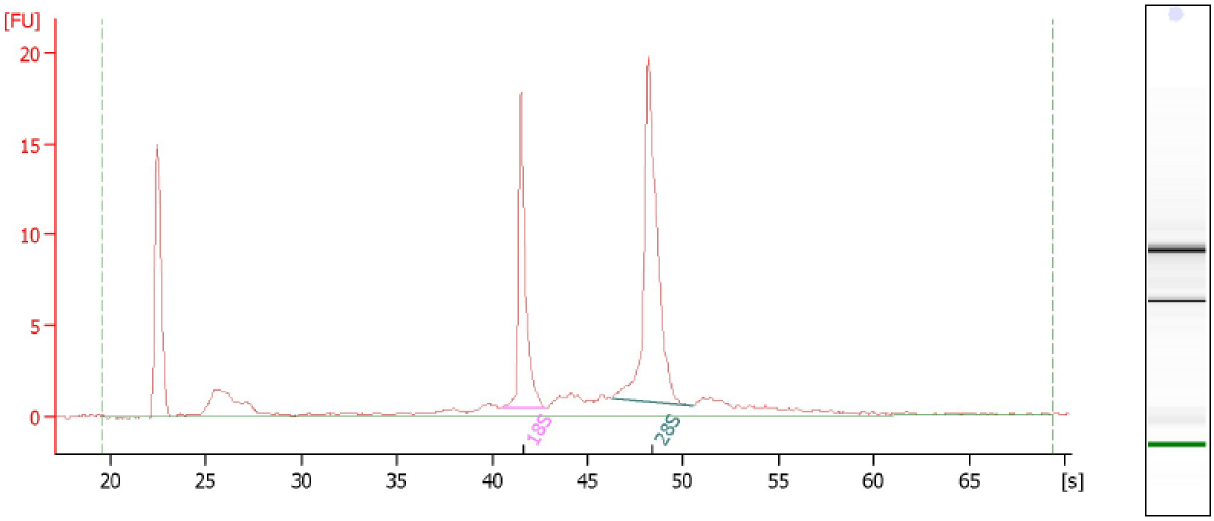

Overall Results for sample 7 : **Sample 7**

RNA Area:	86,6	RNA Integrity Number (RIN):	9.7 (B.02.08)
RNA Concentration:	41 ng/µl	Result Flagging Color:	
rRNA Ratio [28s / 18s]:	1,8	Result Flagging Label:	RIN: 9.70

Fragment table for sample 7 : **Sample 7**

Name	Start Time [s]	End Time [s]	Area	% of total Area
18S	40,48	42,79	17,1	19,7
28S	46,31	50,52	31,5	36,4

Sample 8

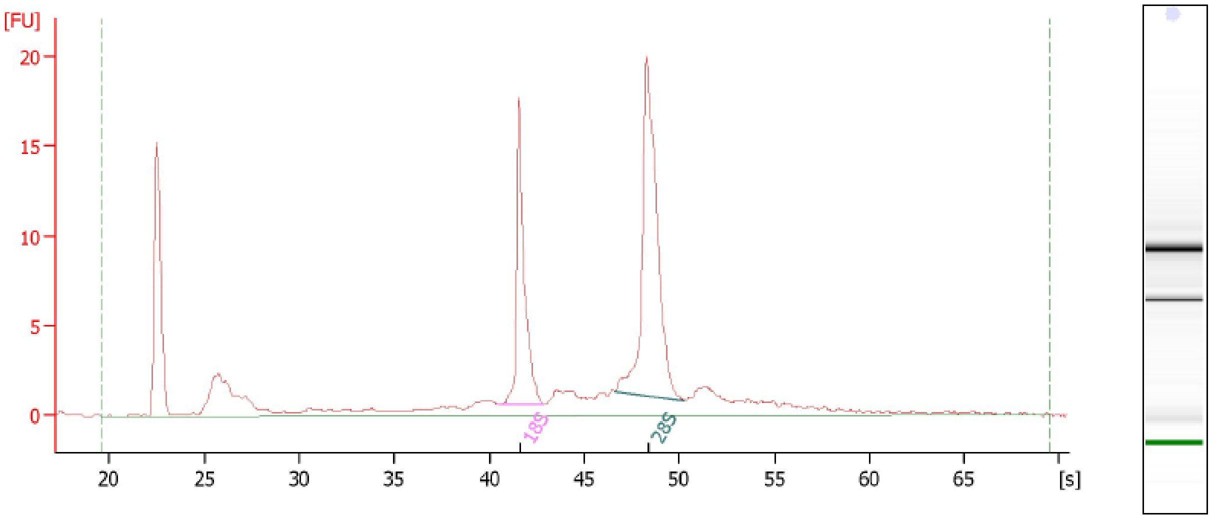

Overall Results for sample 8 : **Sample 8**

RNA Area:	115,5	RNA Integrity Number (RIN):	9.4 (B.02.08)
RNA Concentration:	54 ng/µl	Result Flagging Color:	
rRNA Ratio [28s / 18s]:	1,8	Result Flagging Label:	RIN: 9.40

Fragment table for sample 8 : **Sample 8**

Name	Start Time [s]	End Time [s]	Area	% of total Area
18S	40,47	42,89	19,4	16,8
28S	46,61	50,23	35,2	30,5

Sample 9

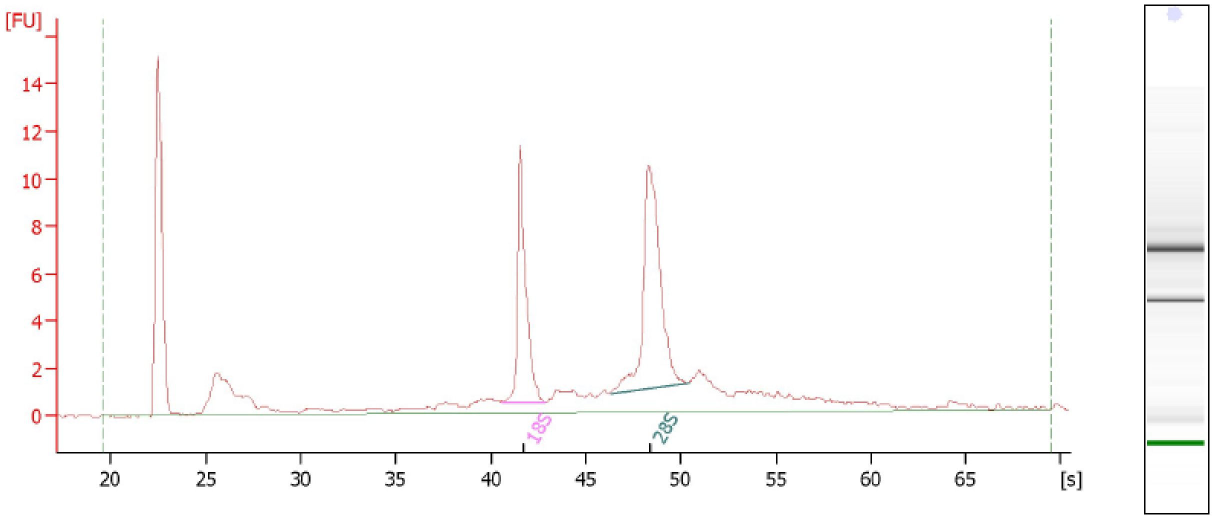

Overall Results for sample 9 : **Sample 9**

RNA Area:	84,7	RNA Integrity Number (RIN):	9.4 (B.02.08)
RNA Concentration:	40 ng/µl	Result Flagging Color:	
rRNA Ratio [28s / 18s]:	1,5	Result Flagging Label:	RIN: 9.40

Fragment table for sample 9 : **Sample 9**

Name	Start Time [s]	End Time [s]	Area	% of total Area
18S	40,62	42,89	13,1	15,5
28S	46,31	50,44	20,1	23,7

Sample 10

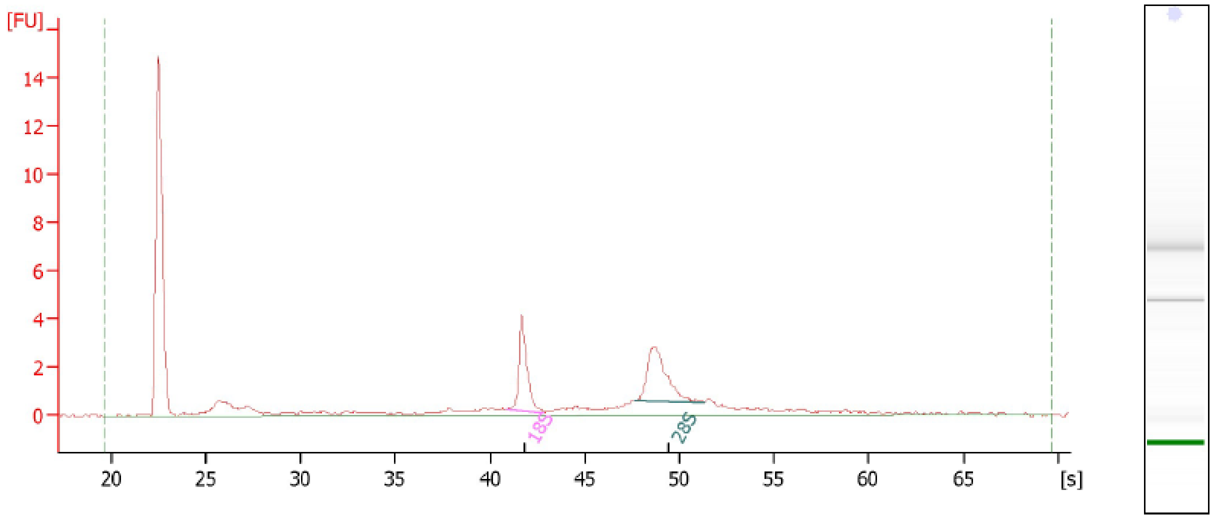

Overall Results for sample 10 : **Sample 10**

RNA Area:	32,5	RNA Integrity Number (RIN):	9.1 (B.02.08)
RNA Concentration:	15 ng/µl	Result Flagging Color:	
rRNA Ratio [28s / 18s]:	1,2	Result Flagging Label:	RIN: 9.10

Fragment table for sample 10 : **Sample 10**

Name	Start Time [s]	End Time [s]	Area	% of total Area
18S	40,91	42,88	4,7	14,5
28S	47,62	51,31	5,6	17,3

Sample 11

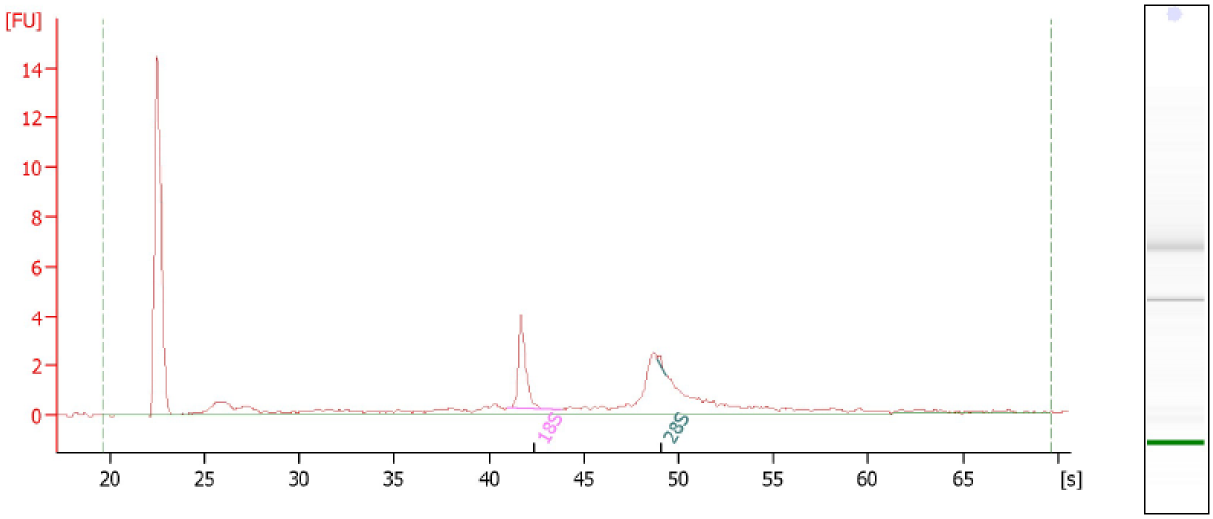

Overall Results for sample 11 : **Sample 11**

RNA Area:	30,8	RNA Integrity Number (RIN):	7.3 (B.02.08)
RNA Concentration:	14 ng/µl	Result Flagging Color:	
rRNA Ratio [28s / 18s]:	0,0	Result Flagging Label:	RIN: 7.30

Fragment table for sample 11 : **Sample 11**

Name	Start Time [s]	End Time [s]	Area	% of total Area
18S	41,01	43,79	4,2	13,6
28S	48,83	49,34	0,2	0,6

Sample 12

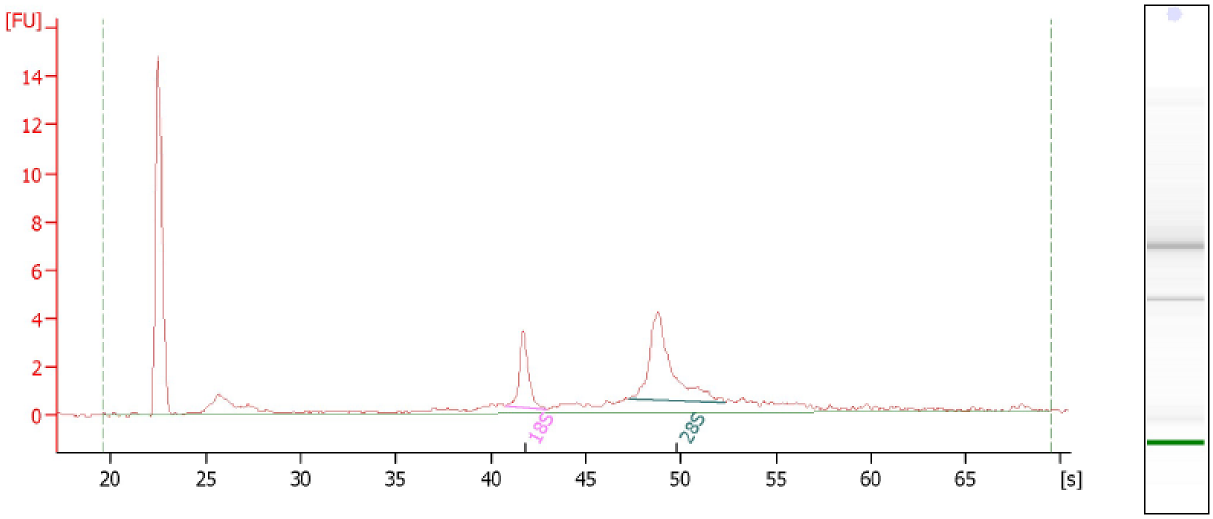

Overall Results for sample 12 : **Sample 12**

RNA Area:	39,0	RNA Integrity Number (RIN):	9.1 (B.02.08)
RNA Concentration:	18 ng/µl	Result Flagging Color:	
rRNA Ratio [28s / 18s]:	2,4	Result Flagging Label:	RIN: 9.10

Fragment table for sample 12 : **Sample 12**

Name	Start Time [s]	End Time [s]	Area	% of total Area
18S	40,82	42,94	4,2	10,7
28S	47,27	52,35	10,0	25,6
