# Supplementary material for: Housekeeping gene validation for RT-qPCR studies on synovial fibroblasts derived from healthy and osteoarthritic patients with focus on mechanical loading
Source: PLoS One. 2019 Dec 6;14(12):e0225790. doi: 10.1371/journal.pone.0225790 (PMC6897414; doi:10.1371/journal.pone.0225790)
Supplement: S2 File — (PDF) [file pone.0225790.s006.pdf]

Supplementary Data 2. Primer specificity evaluation via **(a)** agarose gel and **(b)** melting curve analysis of all used samples (left) and a single sample (right).

a

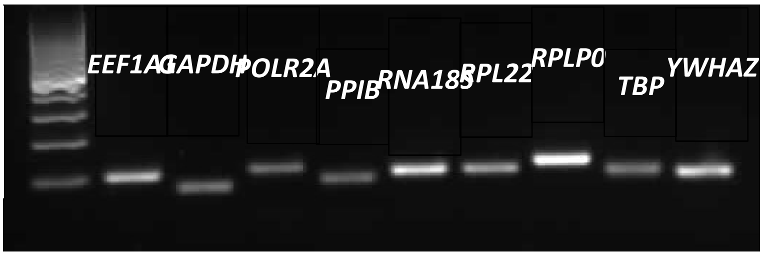

b *EEF1A1*

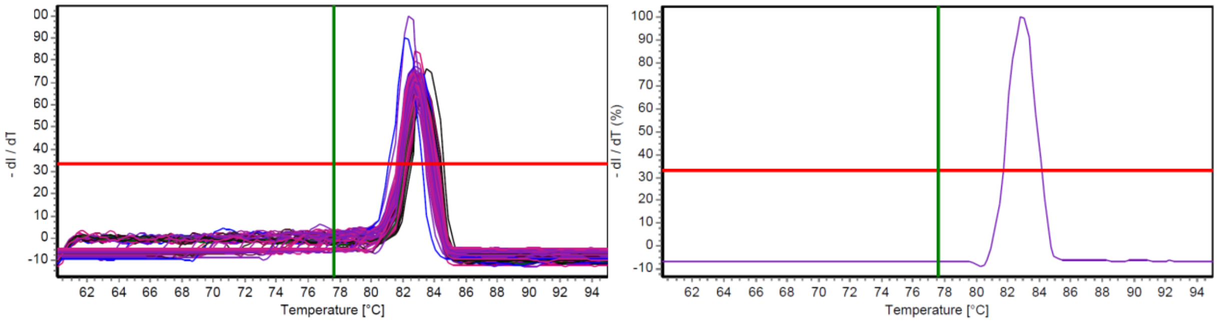

GAPDH

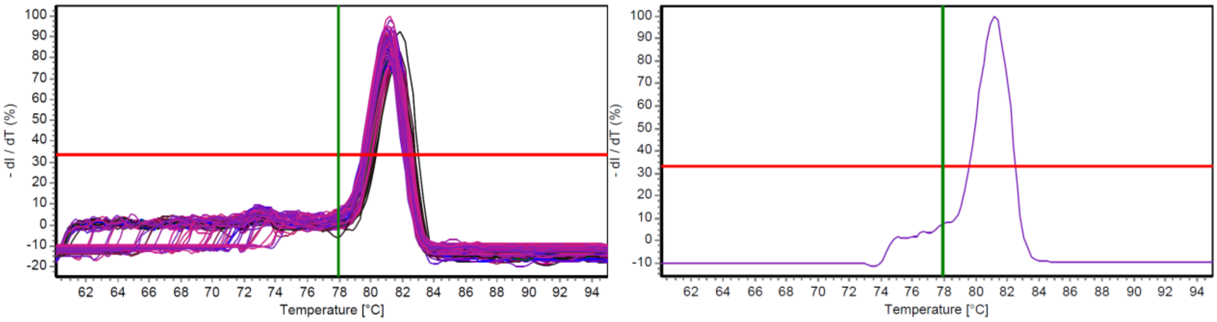

POLR2A

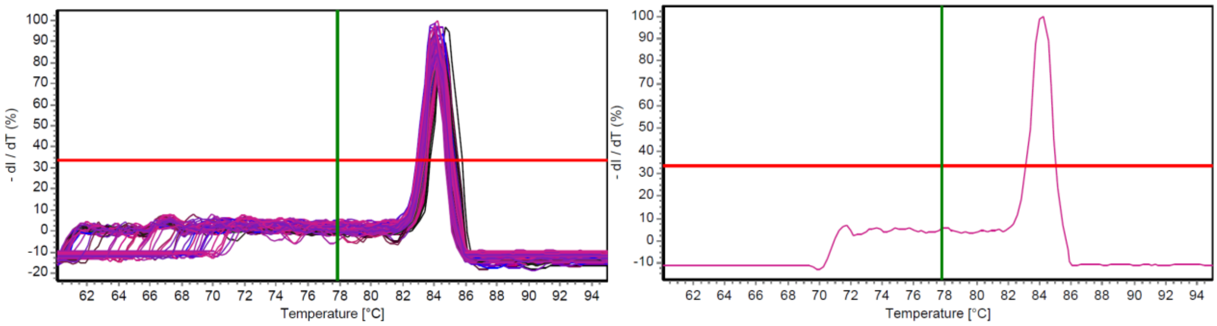

PPIB

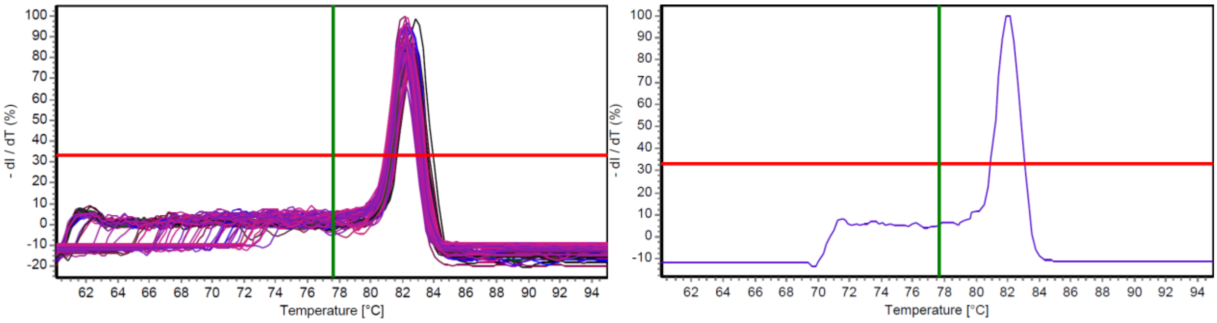

RPL22

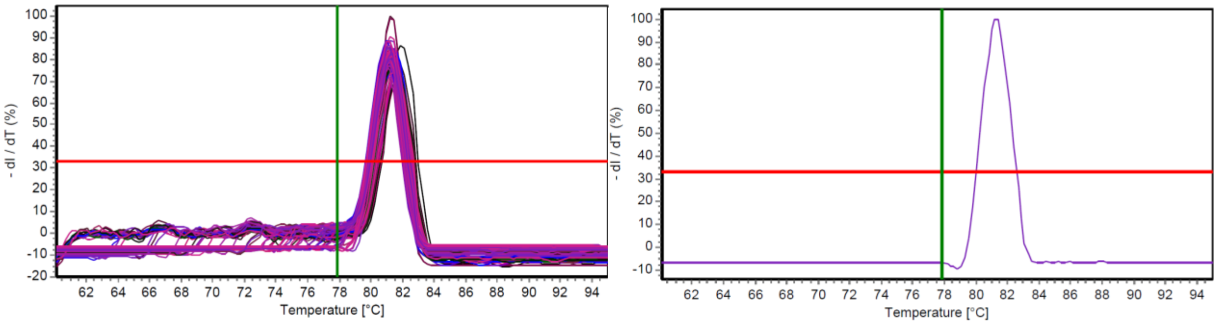

RPLP0

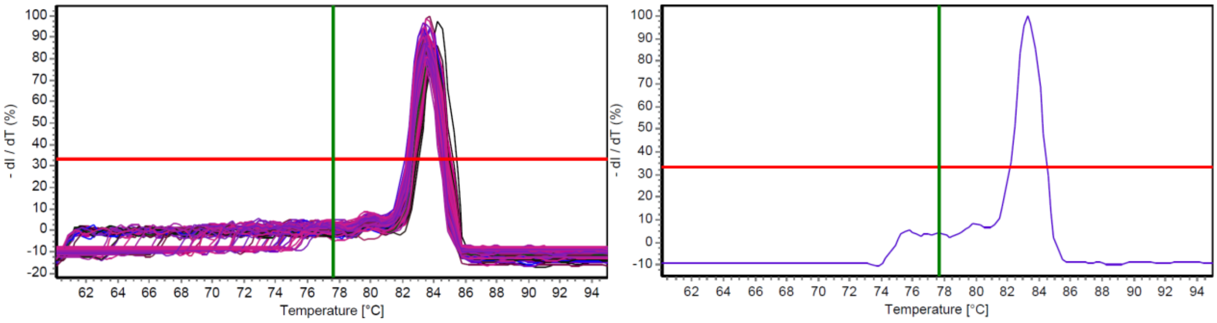

RNA18S

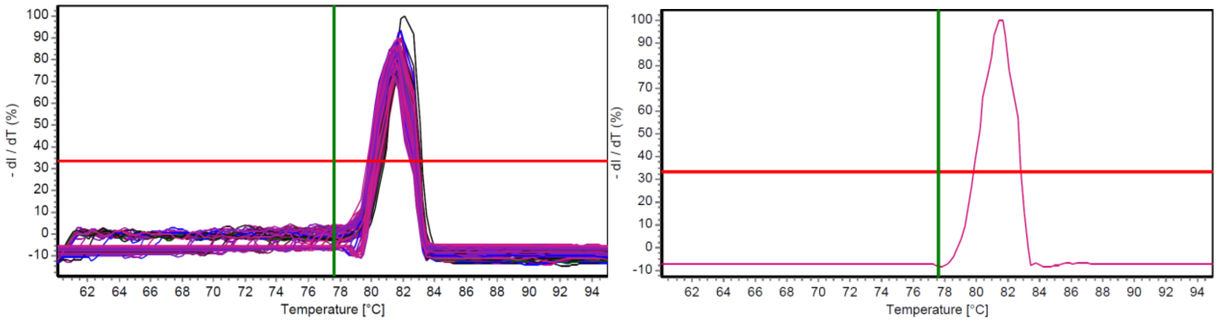

TBP

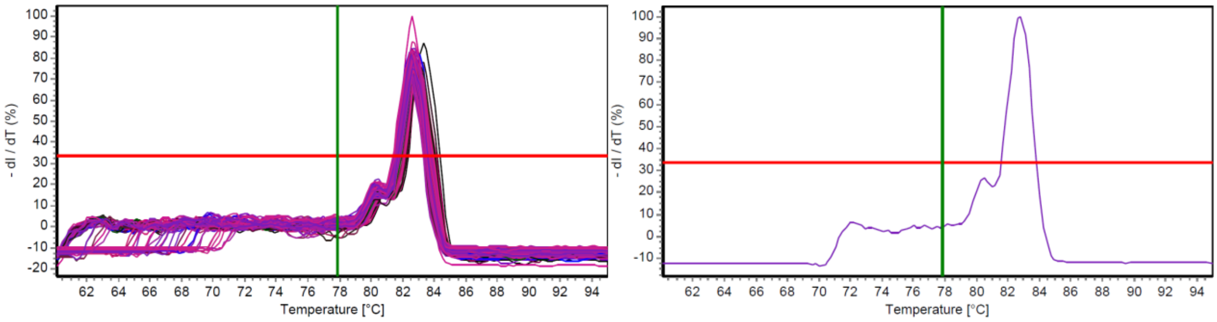
- dI / dT (%)
Temperature [°C]
- dI / dT (%)
Temperature [°C]

YWHAZ

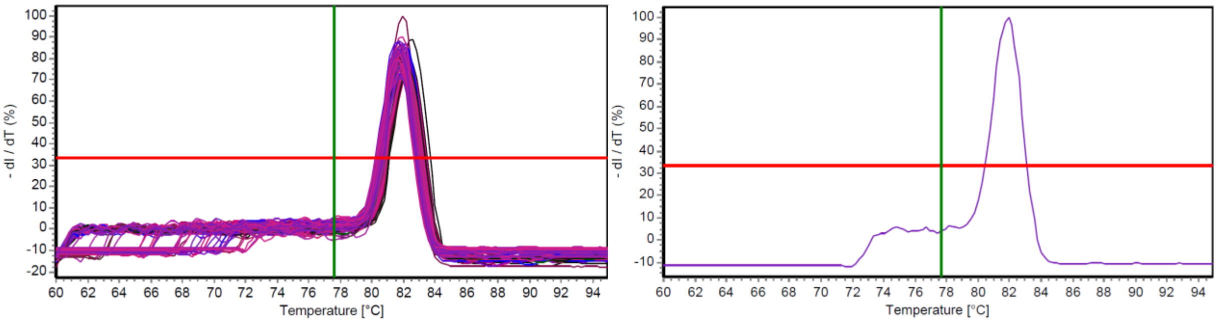
- dI / dT (%)
Temperature [°C]
- dI / dT (%)
Temperature [°C]
