# Supplementary material for: Housekeeping gene validation for RT-qPCR studies on synovial fibroblasts derived from healthy and osteoarthritic patients with focus on mechanical loading
Source: PLoS One. 2019 Dec 6;14(12):e0225790. doi: 10.1371/journal.pone.0225790 (PMC6897414; doi:10.1371/journal.pone.0225790)
Supplement: S3 File — (PDF) [file pone.0225790.s007.pdf]

Collagen-1-alpha-2 mRNA expression relative to

Geometric mean of EEF1A1/RPLP0

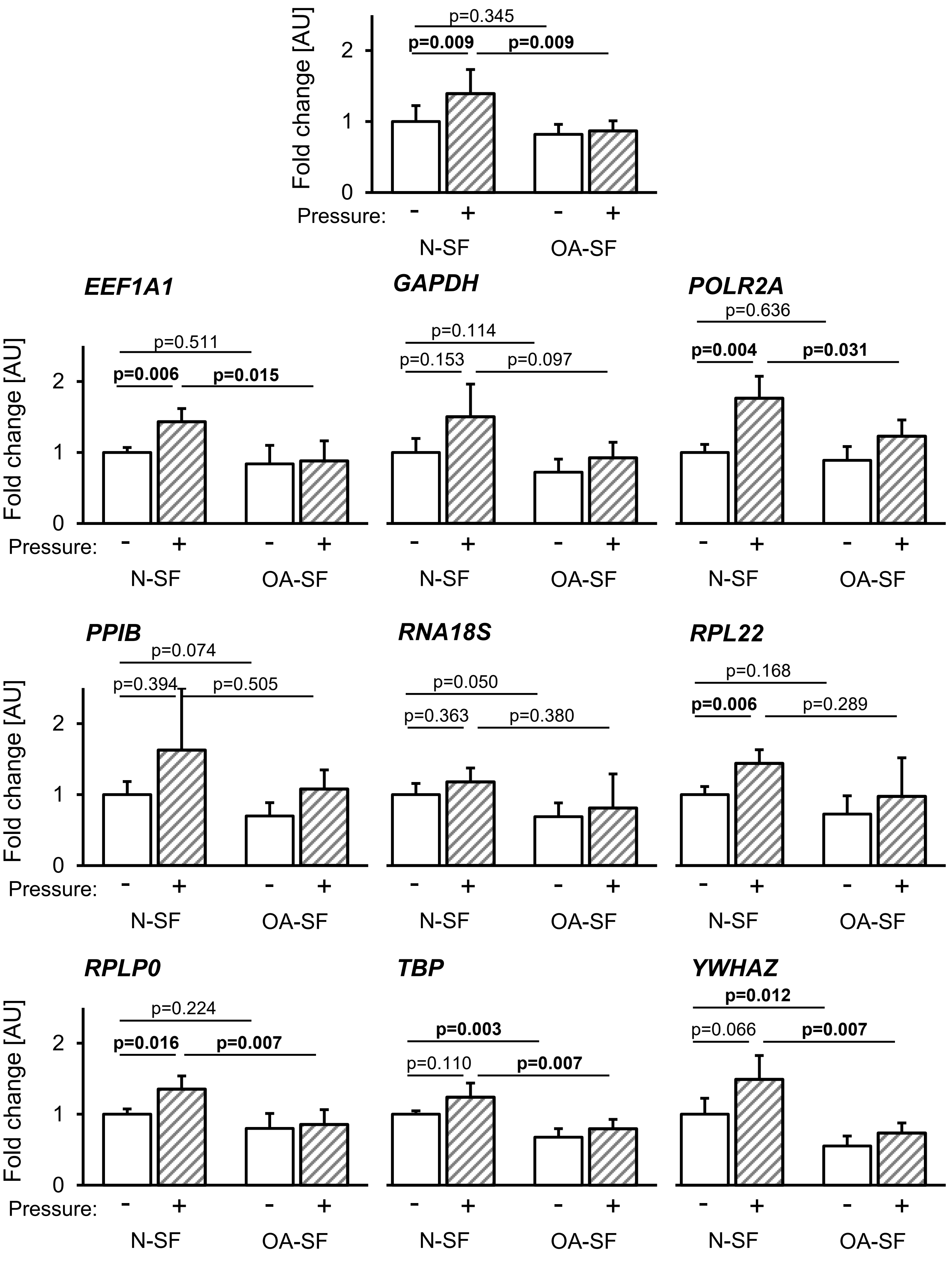

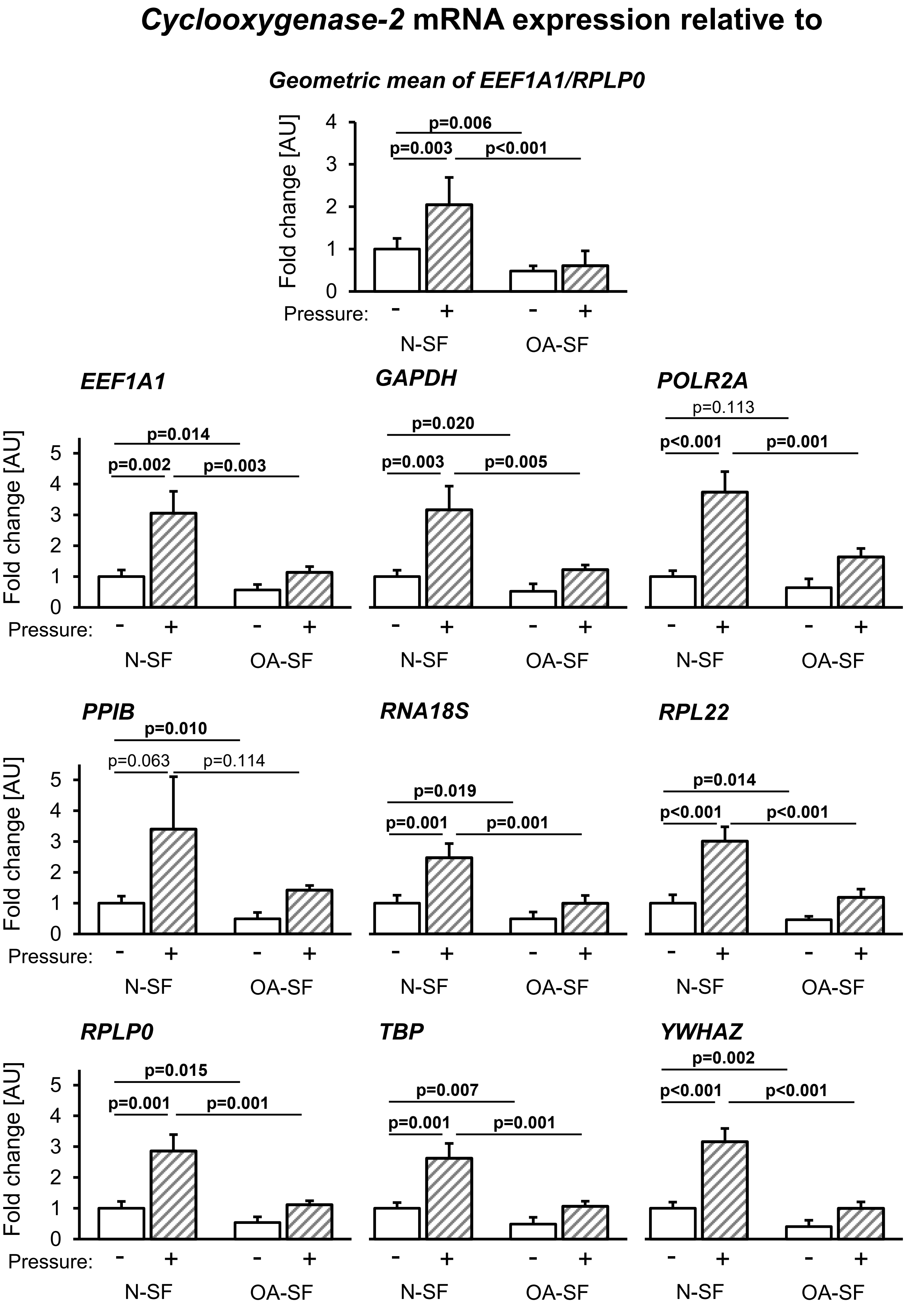
Cyclooxygenase-2 mRNA expression relative to
Geometric mean of EEF1A1/RPLP0
p=0.006
p=0.003
p<0.001
Fold change [AU]
Pressure:
-
+
N-SF
OA-SF
EEF1A1
p=0.014
p=0.002
p=0.003
GAPDH
p=0.020
p=0.003
p=0.005
POLR2A
p=0.113
p<0.001
p=0.001
PPIB
p=0.010
p=0.063
p=0.114
RNA18S
p=0.019
p=0.001
p=0.001
RPL22
p=0.014
p<0.001
p<0.001
RPLP0
p=0.015
p=0.001
p=0.001
TBP
p=0.007
p=0.001
p=0.001
YWHAZ
p=0.002
p<0.001
p<0.001

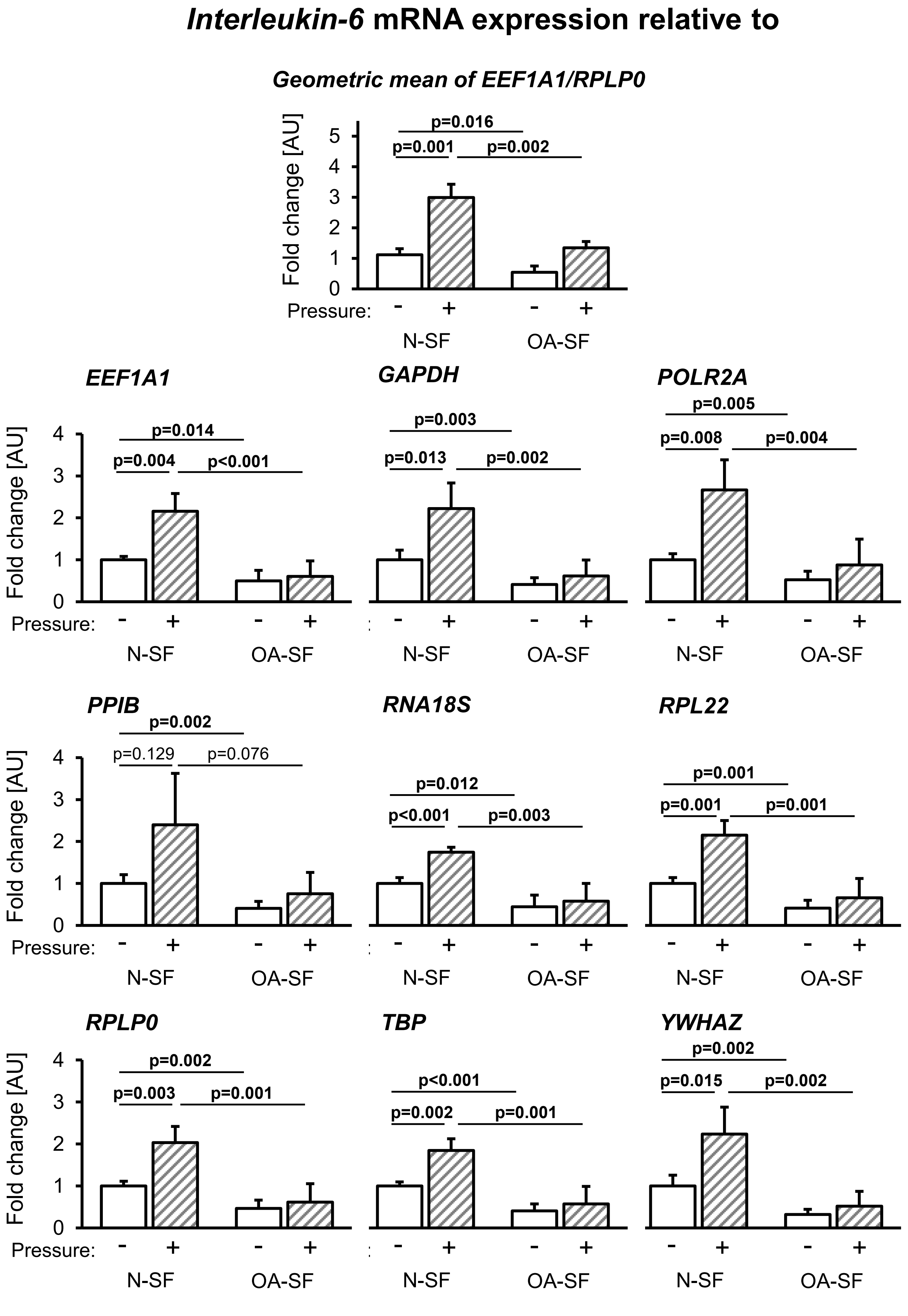
Interleukin-6 mRNA expression relative to
Geometric mean of EEF1A1/RPLP0
p=0.016
p=0.001
p=0.002
Fold change [AU]
Pressure:
N-SF
OA-SF
EEF1A1
p=0.014
p=0.004
p<0.001
GAPDH
p=0.003
p=0.013
p=0.002
POLR2A
p=0.005
p=0.008
p=0.004
PPIB
p=0.002
p=0.129
p=0.076
RNA18S
p=0.012
p<0.001
p=0.003
RPL22
p=0.001
p=0.001
p=0.001
RPLP0
p=0.002
p=0.003
p=0.001
TBP
p<0.001
p=0.002
p=0.001
YWHAZ
p=0.002
p=0.015
p=0.002
